# ﻿Insect herbivore and fungal communities on *Agathis* (Araucariaceae) from the latest Cretaceous to Recent

**DOI:** 10.3897/phytokeys.226.99316

**Published:** 2023-05-26

**Authors:** Michael P. Donovan, Peter Wilf, Ari Iglesias, N. Rubén Cúneo, Conrad C. Labandeira

**Affiliations:** 1 Geological Collections, Gantz Family Collections Center, Field Museum of Natural History, Chicago, IL 60605, USA National Museum of Natural History, Smithsonian Institution Washington United States of America; 2 Department of Paleobotany and Paleoecology, Cleveland Museum of Natural History, Cleveland, OH 44106, USA Geological Collections, Gantz Family Collections Center, Field Museum of Natural History Chicago United States of America; 3 Department of Paleobiology, National Museum of Natural History, Smithsonian Institution, Washington, DC 20013, USA Pennsylvania State University Pennsylvania United States of America; 4 Department of Geosciences, Pennsylvania State University, University Park, Pennsylvania, 16802, USA Cleveland Museum of Natural History Cleveland United States of America; 5 Instituto de Investigaciones en Biodiversidad y Medioambiente, CONICET-Universidad Nacional del Comahue, San Carlos de Bariloche, Río Negro 8400, Argentina Universidad Nacional del Comahue Río Negro Argentina; 6 CONICET-Museo Paleontológico Egidio Feruglio, Trelew, Chubut 9100, Argentina Museo Paleontológico Egidio Feruglio Trelew Argentina; 7 Department of Entomology and Behavior, Ecology, Evolution, and Systematics Program, University of Maryland, College Park, Maryland 20742, USA University of Maryland Maryland United States of America; 8 College of Life Sciences, Capital Normal University, Beijing, 100048, China Capital Normal University Beijing China

**Keywords:** Araucariaceae, Gondwana, herbivory, plant-insect associations

## Abstract

*Agathis* (Araucariaceae) is a genus of broadleaved conifers that today inhabits lowland to upper montane rainforests of Australasia and Southeast Asia. A previous report showed that the earliest known fossils of the genus, from the early Paleogene and possibly latest Cretaceous of Patagonian Argentina, host diverse assemblages of insect and fungal associations, including distinctive leaf mines. Here, we provide complete documentation of the fossilized *Agathis* herbivore communities from Cretaceous to Recent, describing and comparing insect and fungal damage on *Agathis* across four latest Cretaceous to early Paleogene time slices in Patagonia with that on 15 extant species. Notable fossil associations include various types of external foliage feeding, leaf mines, galls, and a rust fungus. In addition, enigmatic structures, possibly armored scale insect (Diaspididae) covers or galls, occur on *Agathis* over a 16-million-year period in the early Paleogene. The extant *Agathis* species, throughout the range of the genus, are associated with a diverse array of mostly undescribed damage similar to the fossils, demonstrating the importance of *Agathis* as a host of diverse insect herbivores and pathogens and their little-known evolutionary history.

## ﻿Introduction

*Agathis* (Araucariaceae) is a genus of broadleaved conifers with ca. 17 extant species that are historically dominant in many areas of lowland to upper montane rainforests from Sumatra to New Zealand (Farjon 2010). The first South American and earliest known members of the genus occur as well-preserved vegetative and reproductive fossils in floras from the early Paleocene (ca. 64 Ma), early and middle Eocene (52.2 and 47.7 Ma), and possibly terminal Cretaceous (66–67 Ma) of Patagonian Argentina (Wilf et al. 2014; Escapa et al. 2018). The Paleocene species, *A.immortalis*, resolves as a stem lineage (Wilf et al. 2014; Escapa et al. 2018), and the approximately 12-million-year younger Eocene species, *A.zamunerae*, belongs to the crown lineage of *Agathis* based on the presence of many of the derived characters of the genus (Wilf et al. 2014; Escapa et al. 2018). Rich insect feeding damage on all the fossil *Agathis* species from Patagonia suggests that the species hosted diverse herbivore communities (Labandeira and Wappler 2023; but also see Root 1973) of specialized insects, as presented earlier in a paper that emphasized leaf mines (Donovan et al. 2020). The abundant damage provides a rare opportunity to study how insect herbivore communities developed on a host genus in the process of evolving its modern characteristics.

The earlier study (Donovan et al. 2020) found similar suites of insect damage, including leaf mines, external foliage feeding, and galls, on Patagonian *Agathis* fossils across four time slices spanning 18 million years from the latest Cretaceous to middle Eocene, and on extant *Agathis*. Two non-exclusive hypotheses were proposed to explain the pattern of persistent damage types on *Agathis* through time (Donovan et al. 2020): The first hypothesis is that similar damage-type morphology represents convergence, possibly due to the relatively unchanged leaf architecture and chemistry of *Agathis*, wherein unrelated groups of insects with similar feeding behaviors colonized *Agathis* repeatedly over time. Second, the evolutionarily conservative morphology and habitat preferences of *Agathis* (Kooyman et al. 2014; Wilf et al. 2014; Merkhofer et al. 2015) may have provided stability for ecological guilds or possibly herbivore communities containing the same insect lineages to persist on the genus over geologic time. A combination of these two scenarios likely contributed to the pattern of multiple persistent associations observed on *Agathis* through time (Donovan et al. 2020). By the early Eocene, diversified, modern-aspect *Agathis* probably ranged throughout Gondwanan rainforest biomes (Wilf et al. 2014). The genus may have been restricted to rainforest environments throughout its history, tracking suitable habitat during changing climates (Kooyman et al. 2014, 2022; Wilf et al. 2014; Merkhofer et al. 2015). Extant *Agathis* leaves have accessory xylem tissues adjacent to the veins (Kausik 1976). These tissues can collapse during periods of drought (Brodribb and Holbrook 2005), which underscores the necessity of elevated moisture availability.

In this study, we provide complete documentation of all the herbivore communities associated with fossil *Agathis* from the latest Cretaceous to middle Eocene of Patagonian Argentina as a follow-up to our previous study that focused on leaf mines (Donovan et al. 2020). We examined insect and fungal damage on cf. *Agathis* fossils from the latest Cretaceous part of the Lefipán Formation, *Agathisimmortalis* from the early Paleocene (early Danian) Salamanca Formation, and *Agathiszamunerae* from the early Eocene Laguna del Hunco and middle Eocene Río Pichileufú sites (Huitrera Formation). Together, these assemblages represent four time slices spanning discontinuously an approximately 18 million-year-long interval from the latest Cretaceous to middle Eocene in which the evolution of the herbivore communities on *Agathis* was examined. We also document representative insect and pathogen damage on 15 species of extant *Agathis* from herbarium collections for comparisons with the fossils. We provide detailed descriptions of insect and fungal damage and discuss the biology of these associations and their extant analogs.

## ﻿Methods

We compared insect and fungal damage on Patagonian *Agathis* fossils (482 specimens), including isolated leaves, leafy branches, and cone scales, from four latest Cretaceous to early middle Eocene fossil assemblages described below. We described all insect and fungal damage, except for previously described blotch mines (Donovan et al. 2020). Damage type numbers (DTs) were assigned with the “Guide to Insect (and Other) Damage Types on Compressed Fossil Plants” (Labandeira et al. 2007) and subsequent published and unpublished supplements. *Agathis* fossils from the Late Cretaceous Lefipán Formation, the early Paleocene Palacio de los Loros 2 (PL2) site, and the early Eocene Laguna del Hunco (LH) flora are housed at the Museo Paleontológico Egidio Feruglio (**MPEF-Pb**) in Trelew, Chubut Province, Argentina. Recent Río Pichileufú (RP) collections are curated at the Museo Paleontológico Bariloche (**BAR**), San Carlos de Bariloche, Río Negro Province (examined while on loan at MPEF), and the type material from RP first published by Berry (1938, under the name “*Zamiatertiaria*”) is housed at the Smithsonian Institution, National Museum of Natural History (**USNM**), in Washington, DC, United States of America. Specimen numbers beginning with LefE, LefL, LefW, and PL2 are unique field numbers not yet assigned repository numbers, and these specimens are curated at MPEF.

The Lefipán Formation is a tidally dominated delta deposit in northwest Chubut, Argentina (Scasso et al. 2012; Vellekoop et al. 2017), which preserves a diverse Maastrichtian macroflora and Maastrichtian–Danian microfloras and marine invertebrates (67–66 Ma based on biostratigraphic age constraints; Kiessling et al. 2005; Barreda et al. 2012; Scasso et al. 2012; Vellekoop et al. 2017). As detailed elsewhere (Scasso et al. 2012; Donovan et al. 2017), three fossil plant localities (LefE, LefL, LefW) are located in close proximity. LefE and LefW are located approximately 1000 m from each other on opposite sides of a ridge, and LefL is ~500 m to the east of LefE at the same stratigraphic level. Although no reproductive organs or in situ cuticles have been found, cf. *Agathis* leaves (10 specimens) from the Lefipán Formation exhibit characters associated with extant members of the genus, including a symmetrical, lanceolate shape, parallel venation, and a short, constricted petiole. The Lefipán flora is dominated by angiosperms (Stiles et al. 2020; Cunéo et al. 2021) and associated with diverse insect damage (Donovan et al. 2017, 2018). Besides likely *Agathis*, other conifers from the formation include *Araucarialefipanensis* (Araucariaceae; Andruchow-Colombo et al. 2018), *Patagotaxodialefipanensis* (Cupressaceae; Andruchow-Colombo et al. 2022), and *Retrophyllumsuperstes* (Podocarpaceae; Wilf et al. 2017).

Danian *Agathisimmortalis* Escapa, Iglesias, Wilf, Catalano, Caraballo, and Cúneo fossils (319 specimens) are from Palacio de los Loros 2 (PL2), a fossil plant locality in the estuarine Salamanca Formation in southern Chubut (Iglesias et al. 2007, 2021; Stiles et al. 2020) deposited during chron C28n (64.67–63.49 Ma) (Gradstein et al. 2012; Clyde et al. 2014; Comer et al. 2015). *Agathisimmortalis* specimens from PL2 (leaves, cone scales, winged seeds, and pollen cones with in situ pollen) have similar morphology to extant and fossil *Agathis*, with reproductive features that suggest a basal position within the lineage (Escapa et al. 2018). The PL2 flora is angiosperm dominated (Iglesias et al. 2007, 2021), and other well-described elements of the flora include Cunoniaceae flowers (Jud et al. 2018a; Jud and Gandolfo 2021), Menispermaceae endocarps and leaves (Jud et al. 2018b), a podocarpaceous conifer (Andruchow-Colombo et al. 2019), and *Azolla* (Salviniaceae) sporophytes (Hermsen et al. 2019).

*Agathiszamunerae* Wilf fossils (Wilf et al. 2014), including leaves, leafy branches, pollen cones, and cone scales with in-situ seeds, occur at two Eocene caldera-lake deposits in the Huitrera Formation, Laguna del Hunco (LH; 121 specimens; ^40^Ar-^39^Ar dated tuff from the fossiliferous interval with age 52.22 ± 0.22 Ma; Wilf et al. 2005b; Wilf 2012) and Río Pichileufú (RP; 32 specimens; ^40^Ar-^39^Ar dated tuff immediately above the main fossiliferous horizon of 47.74 ± 0.05 Ma; Wilf et al. 2005b; Wilf 2012), located in northwest Chubut and western Río Negro provinces, Argentina, respectively. Numerous paleontological studies have been conducted on fossil plants, insects, frogs, and fish from these localities, as summarized by Wilf et al. (2013, 2014) and (Barreda et al. 2020).

We also surveyed insect and fungal damage on extant *Agathis* specimens from several herbaria (in person), including nearly all *Agathis* collections at the Arnold Arboretum Herbarium (**A**) and Gray Herbarium (**GH**) of the Harvard University Herbaria, Royal Botanic Garden Edinburgh (**E**), Royal Botanic Gardens Kew (**K**), United States National Herbarium (**US**), Australian National Herbarium (**CANB**), National Herbarium of New South Wales (**NSW**), and the Singapore Botanic Gardens Herbarium (**SING**). From these collections, we documented representative insect and pathogen damage on 15 species of *Agathis*. Extant leaf mines associated with *Agathis* were covered in depth in the supplement of Donovan et al. (2020) and are re-illustrated and briefly described in the text here for the sake of completeness. Extant species taxonomy followed Farjon (2010).

Macro- and microphotographic methods for fossil and extant specimens were detailed previously (Donovan et al. 2020). We applied DT keywords to specimen photographs with Adobe Bridge to facilitate rapid comparisons between fossil and extant specimens (Rossetto-Harris et al. 2022). We used Adobe Photoshop CC 2017 to compose images and Adobe Camera Raw Editor to change temperature, white balance, contrast, and other features on whole images as needed.

## ﻿Results: Insect herbivory on fossil *Agathis*

### ﻿Latest Cretaceous, Lefipán Formation

**Figure 1.**
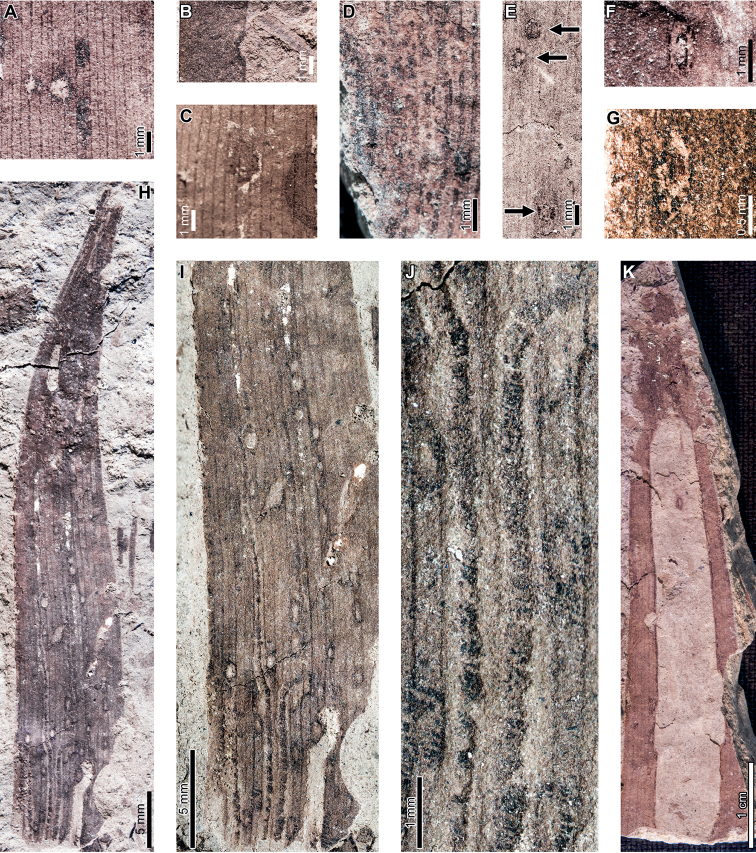
Insect damage on cf. *Agathis* leaves from the Lefipán Formation **A** circular hole with dark reaction rim (DT1; MPEF-Pb 9835) **B** small excision into the leaf margin (DT12) **C** patches of surface feeding (DT29) **D** cluster of circular piercing and sucking marks (DT46; MPEF-Pb 9837) **E** dark circular galls (DT32; MPEF-Pb 9829) **F** elliptical oviposition mark (DT101; MPEF-Pb 9841) **G** elliptical oviposition mark (DT101) **H** linear serpentine mines following leaf venation (DT139; MPEF-Pb 9836) **I** close-up of mines in (**H**) **J** detail of frass trail in (**H**) **K** oblong blotch mine (DT88; MPEF-Pb 9839).

Latest Cretaceous (Maastrichtian) cf. *Agathis* leaves from the Lefipán Formation are preserved with hole feeding, margin feeding, surface feeding, piercing and sucking, mining, galling, and oviposition damage. External foliage feeding (Fig. 1A–C) includes circular holes measuring 0.4–1.0 mm in diameter (DT1; Fig. 1A). The holes are surrounded by a 0.1–0.2 mm wide reaction rim, and the margins of the holes are not influenced by leaf venation. A shallow, semicircular excision into the leaf margin (DT12; Fig. 1B) measures 1.1 mm wide by 0.4 mm deep with a 0.3 mm wide reaction rim. Circular to polylobate patches of surface feeding (DT29; Fig. 1C) measure 1.4–3.9 mm in diameter.

A cluster of black marks probably represents piercing-and-sucking damage (DT46; Fig. 1D). The marks, or punctures, measure 0.1–0.2 mm in diameter. Some of these punctures are composed of a black rim that surrounds lighter tissue, interpreted as a reaction rim surrounding a pinpoint feeding site.

An oblong blotch mine lacking frass, occurring along the central axis of a leaf, was previously described by Donovan et al. 2020 (Fig. 1K). A serpentine mining association, characterized by a linear path largely confined by parallel venation (DT139; Fig. 1H–J), is also associated with a cf. *Agathis* leaf. The mine varies in width between 0.5–0.7 mm with no obvious directional width increase. The origin and overall trajectory of the mine, or possibly multiple mines, is difficult to discern because the entire leaf was not recovered, and some detail is obscured by poor preservation (Fig. 1I). Linear mine paths lie between adjacent parallel veins (Fig. 1J), although the mine sporadically crosscuts or straddles the veins. The central frass trail measures 0.3–0.4 mm wide (35–80% of the mine width) and is densely packed. The moderately to tightly sinusoidal frass trail is intermittent and may consist of spheroidal pellets. The lateral margins of the mine are smooth and parallel-sided (Fig. 1J).

Carbonized, oval galls measure 1.0–1.1 mm in maximum diameter by 0.8–0.9 mm in minimum diameter (DT32; Fig. 1E). The long axes of the galls are parallel to the leaf veins. The galls have a slight positive relief relative to the leaf surface.

Oviposition lesions are composed of inner elliptical, disturbed tissues surrounded by scar tissue, such as callus (Fig. 1F, G). The inner disturbed tissue areas measure 0.6 mm long by 0.2 mm wide, and the reaction tissue is 0.1–0.4 mm wide. The oviposition lesions are oriented parallel to major venation.

### ﻿Early Paleocene, Palacio de los Loros 2

**Figure 2.**
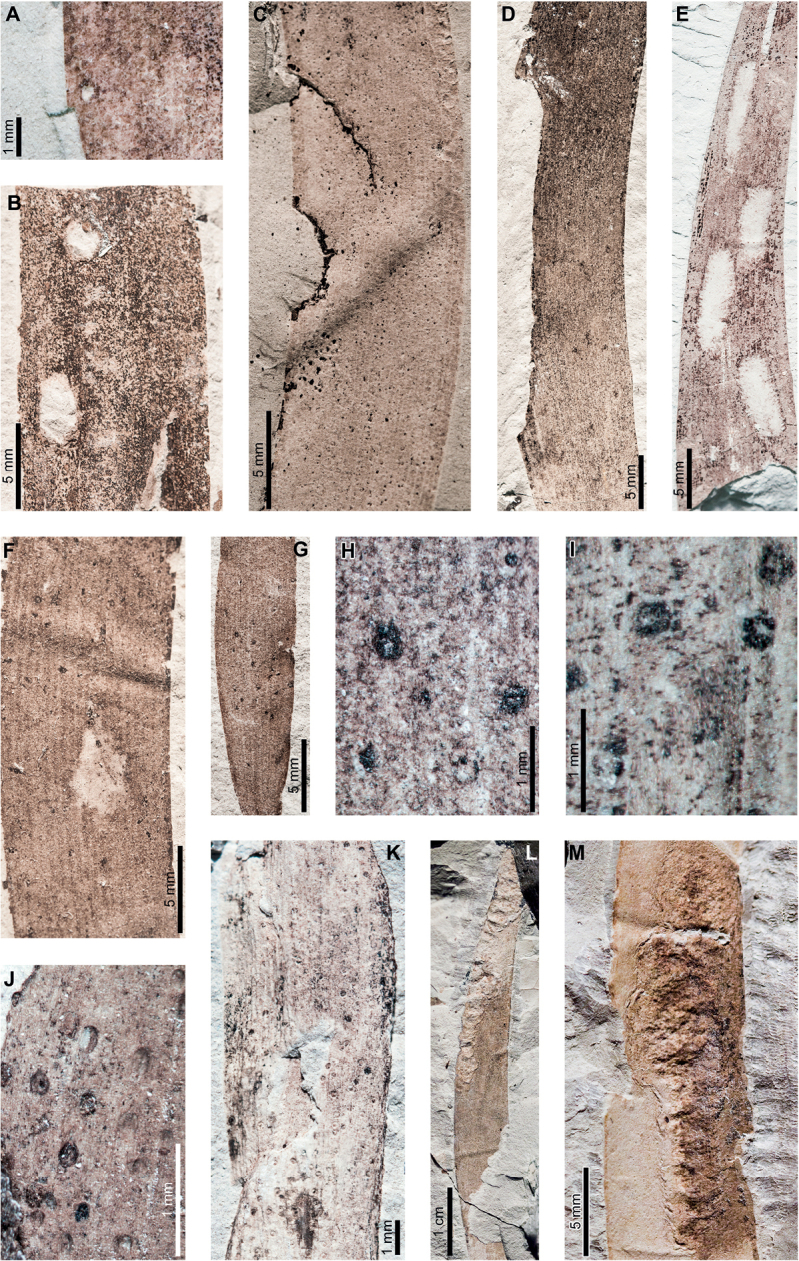
External foliage feeding, piercing and sucking, and leaf mining on *Agathisimmortalis* from Palacio de los Loros 2 **A** small, circular hole (DT1; MPEF-Pb 6010) **B** circular and elliptical holes (DT2; MPEF-Pb 6042) **C** semicircular excision into the leaf margin (DT12; MPEF-Pb 6091) **D** elongate excision into the leaf margin (DT12; MPEF-Pb 9768) **E** zones of surface feeding (DT29; MPEF-Pb 9774) **F** patch of surface feeding (DT29; MPEF-Pb 6030) **G** cluster of piercing and sucking marks (DT46; MPEF-Pb 9766) **H** detail of piercing and sucking marks in (**G**) with central depressions **I** detail of piercing and sucking marks in (**G**) (DT46; MPEF-Pb 5959) **J** detail of piercing and sucking marks with central depressions in (**K**) (DT46; MPEF-Pb 9779) **K** clusters of piercing and sucking marks (DT46; MPEF-Pb 5959) **L***Frondicuniculumflexuosum* blotch mine (DT421; paratype MPEF-Pb 6001) **M***F.flexuosum* blotch mine (DT421; holotype MPEF-Pb 5970).

Insect feeding on early Paleocene (Danian) *Agathisimmortalis* at PL2 in the Salamanca Formation includes hole feeding, margin feeding, surface feeding, piercing and sucking, mining, and galling. External foliage feeding (Fig. 2A–F) includes circular holes measuring 0.3–3.4 mm in diameter (DT1, DT2; Fig. 2A, B) surrounded by 0.2 mm wide reaction rims. Shallow, approximately circular excisions into the leaf margin (DT12; Fig. 2C, D) measure 5.2–33.0 mm long by 1.0–1.8 mm deep into the leaf blade. Reaction tissue surrounding the excisions measures 0.1–0.3 mm wide. Circular to oblong surface feeding zones lack reaction rims (DT29; Fig. 2E, F) and measure 5.0–8.5 mm long by 1.3–2.7 mm wide. Leaf veins within the surface feeding zones are faintly visible or locally not visible.

Circular piercing-and-sucking marks (DT46; Fig. 2G–K) measure 0.1–0.3 mm in diameter. Some punctures have a depression at their centers (Fig. 2H–J). The punctures are typically clustered on a leaf.

*Agathisimmortalis* is associated with *Frondicuniculumflexuosum*, an elongate-ellipsoidal blotch mine with undulatory margins with a wrinkled appearance (DT421; Donovan et al. 2020). The mines are positioned along leaf margins with their long-axes parallel to leaf veins. Frass is deposited as distinct pellets or more amorphous packets, either along one margin of the mine or throughout (Fig. 2L, M). We assign *F.flexuosum* to DT421 (Suppl. material 1) for use in future versions of the “Guide to Insect (and Other) Damage Types on Compressed Fossil Plants” (Labandeira et al. 2007), and detailed descriptions and an ichnotaxonomic treatment of *F.flexuosum* were provided by Donovan et al. 2020.

**Figure 3.**
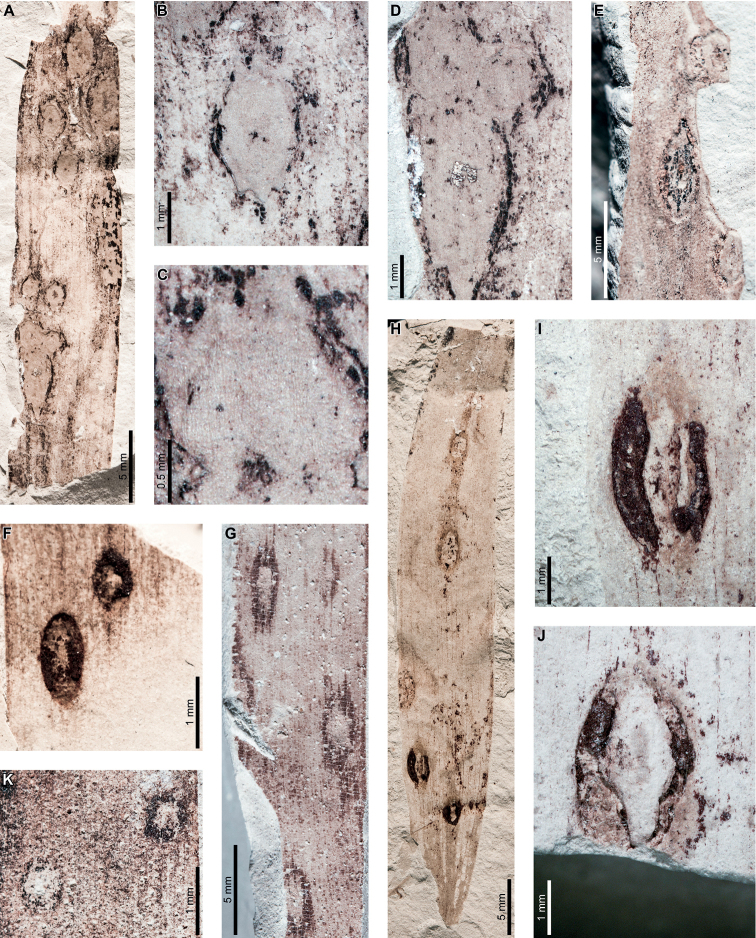
Galls on *Agathisimmortalis* from Palacio de los Loros 2 (**A–K**) **A** elliptical galls with thickened outer walls surrounding epidermal tissue (DT115; MPEF-Pb 9767) **B** detail of oval gall with thickened walls in (**A**) **C** detail of gall in (**B**) showing files of epidermal cells and a dot representing the larval chamber or exit hole **D** detail of cluster of elliptical and circular galls in (**A**) **E** elliptical gall covered in carbonized, thickened tissue (DT115; MPEF-Pb 5977) **F** galls with thickened outer walls and central larval chamber (DT115; MPEF-Pb 6029) **G** galls with thickened outer walls (DT115; MPEF-Pb 6027) **H** five galls with carbonized, thickened walls (DT115; MPEF-Pb 9773) **I** detail of elliptical gall with carbonized, thickened walls in (**H**) **J** detail of elliptical gall with carbonized, thickened walls in (**H**) **K** circular galls with thickened outer tissue surrounding unthickened inner area (DT11; MPEF-Pb 5983).

*Agathisimmortalis* is associated with three gall DTs. Distinctive ellipsoidal to near-circular galls with thickened walls (DT115; Fig. 3A–J) measure (1.0) 2.0–5.0 (9.9) mm long by (0.9) 1.5–3.0 (3.9) mm wide. The galls are oriented with their long axes parallel to the leaf veins. The outer walls of the galls are composed of a thickened layer of woody tissue (Fig. 3I, J) preserved as carbonized material and measuring 0.2–0.9 mm wide. The hardened outer wall surrounds unthickened tissue where the internal chamber was located. The galls are situated on epidermal tissue, and epidermal cells are visible in the center of some galls (Fig. 3B–D). However, leaf veins are not visible within the galls. For those galls that preserve epidermal tissue, a small, black splotch, possibly representing the position of the larval chamber or an exit hole, is located near the center (Fig. 3B–D). The thickened outer rims of the galls may have extended over the entire surfaces of these structures (Fig. 3E). Circular galls with an outer rim of thickened tissue surrounding a zone of unthickened tissue (DT11; Fig. 3K) measure 0.7–1.6 mm in diameter. The thickened rim, 0.2–0.4 mm wide, is striated and carbonized. Although common at PL2, this gall type is not found on *Agathis* at any other locality.

**Figure 4.**
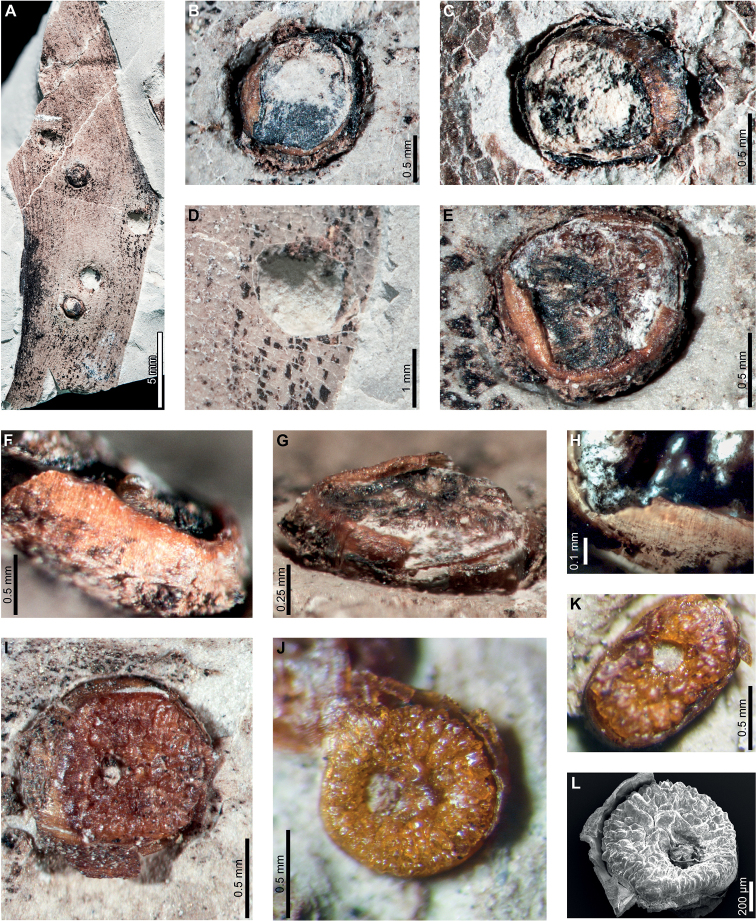
Galls (DT116) on *Agathisimmortalis* from Palacio de los Loros 2 **A** columnar galls and pits where galls were not preserved (MPEF-Pb 6023) **B** detail of gall in (**A**) **C** columnar gall with striated side (MPEF-Pb 6088) **D** detail of pit in (**A**) **E** columnar scale with striated side overlapping the top (MPEF-Pb 5995) **F** side view of gall in (**E**) showing horizontal and vertical striations on the ventral cover **G** side view of gall in (**E**) **H** detail of gall in (**E**) under epifluorescence showing vertical and horizontal striations **I** columnar gall (MPEF-Pb 5960) **J** columnar gall preserved as amber (MPEF-Pb 9750) **K** columnar gall preserved as amber (MPEF-Pb 9750) **L** SEM image of gall in (**J**) showing texture (MPEF-Pb 9750).

The third gall DT associated with *Agathisimmortalis* is defined as a columnar gall protruding above the leaf surface (DT116; Fig. 4A–L). The galls measure 1.1–1.8 mm in diameter and are ornamented with rounded or pointed bumps (Fig. 4E–K), which appear to be arranged concentrically. Some galls have a substantial oval depression near their center (Fig. 4I–L), which may be a feature of the standard morphology of the galls, analogous to extant galls made by *Neuroterusnumismalis* (Hymenoptera, Cynipidae) on oak leaves (Jankiewicz et al. 2017), an exit hole, or a fungal ostiole. Each gall is surrounded by a thin rim of tissue (Fig. 4F–H), which wraps around the top of the gall on some specimens. The tissue rims measure 0.02–0.12 mm wide and approximately 0.3–0.4 mm tall, marked by horizontal and vertical striations (Fig. 4F–H). These columnar galls appear to be deeply set in the leaf tissue and result in a concave pit when removed (Fig. 4A, D). Most specimens are replaced by or filled in with amber derived from ambient leaf resins.

**Figure 5.**
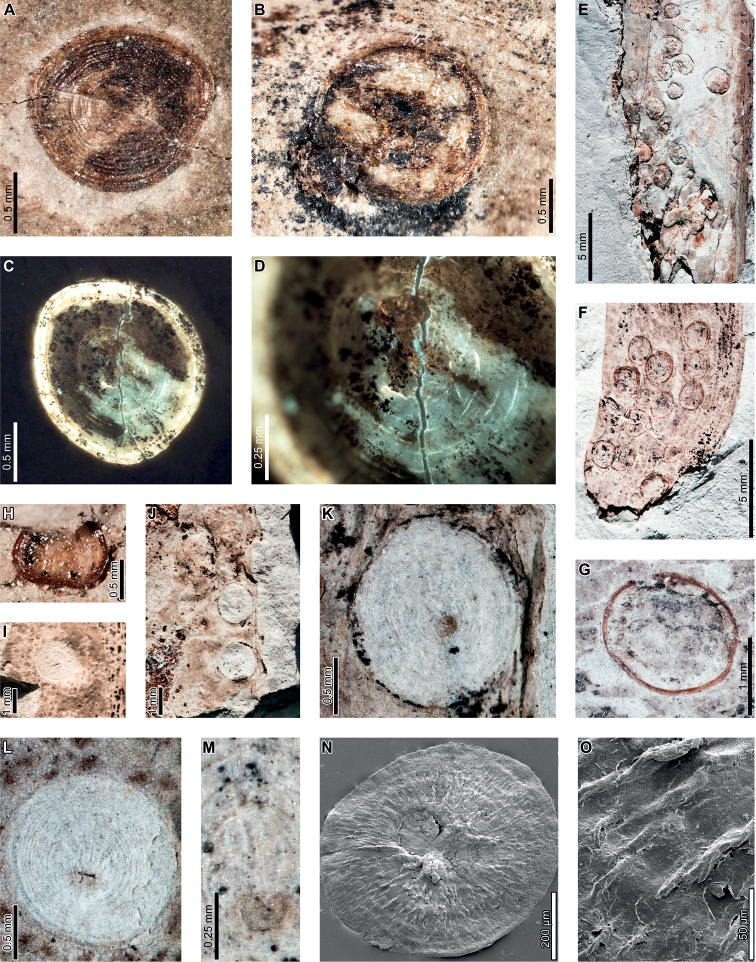
Enigmatic structures, possibly armored scale insect (Diaspididae) covers, (DT86) on *Agathis* leaves (**A–G, I–M**) and a cone scale (**H**) from Palacio de los Loros 2 **A** cover with concentric growth rings (MPEF-Pb 6096) **B** cover with concentric growth rings (MPEF-Pb 6096) **C** cover in (**A**) under epifluorescence **D** detail of (**C**) showing concentric growth rings **E** clusters of cover impressions (MPEF-Pb 6113) **F** embedded ventral cover (MPEF-Pb 6020) **G** embedded ventral cover (MPEF-Pb 5985) **H** cover preserved as amber (MPEF-Pb 5861) **I** impression of cover (MPEF-Pb 5996) **J** impressions of two covers (MPEF-Pb 5996) **K** detail of upper cover impression in (**J**) **L** detail of cover impression in (**I**) **M** detail of lower cover in (**J**) showing concentric growth rings **N** cover showing rod-like structures (MPEF-Pb 9750) **O** close-up of rod-like structures in (**N**).

Enigmatic structures possibly representing armored scale-insect covers (Diaspididae) are associated with leaves (Fig. 5A–G, I–O) and a cone scale (Fig. 5H). The structures, which we refer to as “covers” in the description, may be preserved as amber casts of the dorsal cover and/or ventral cover or “collar” (Fig. 5A–D, F, G, H); impressions of the dorsal covers (Fig. 5E, F, I–M); or impressions where the ventral covers were once located. The dorsal covers are approximately circular to oval, more or less flattened, marked with concentric growth rings, and are surrounded by an attached ventral cover (DT86). Dorsal covers measure 0.87–1.65 mm in maximum diameter by 0.77–1.5 mm in minimum diameter. Concentric growth rings on dorsal covers (Fig. 5A, C, D, K–N) are spaced 0.02–0.08 mm apart. The concentric rings may represent instar growth increments, although the boundaries between the first and second instar and adult phases of the dorsal covers are unclear. An ovoidal bump or pustule (Fig. 5K–N) near the center of the covers in the area presumably made by the first instar nymph may be present, possibly marking the location of the first instar exuviae or the position of stylets from the piercing-and-sucking insects into the subjacent targeted tissues. The ovoidal bumps measure 0.17–0.20 by 0.13–0.16 mm in dimension. On one specimen, rod-like structures radiate from the center of the cover (Fig. 5N, O). The ventral covers that surround the dorsal covers measure 0.06–0.20 mm wide and typically have greater relief than the surrounding unaffected area. The covers share similarities to the galls illustrated in Fig. 4, including the ventral collar and concentric rings, and ovoidal bump or hole on the dorsal side, suggesting that these structures may be related.

### ﻿Early Eocene, Laguna del Hunco

**Figure 6.**
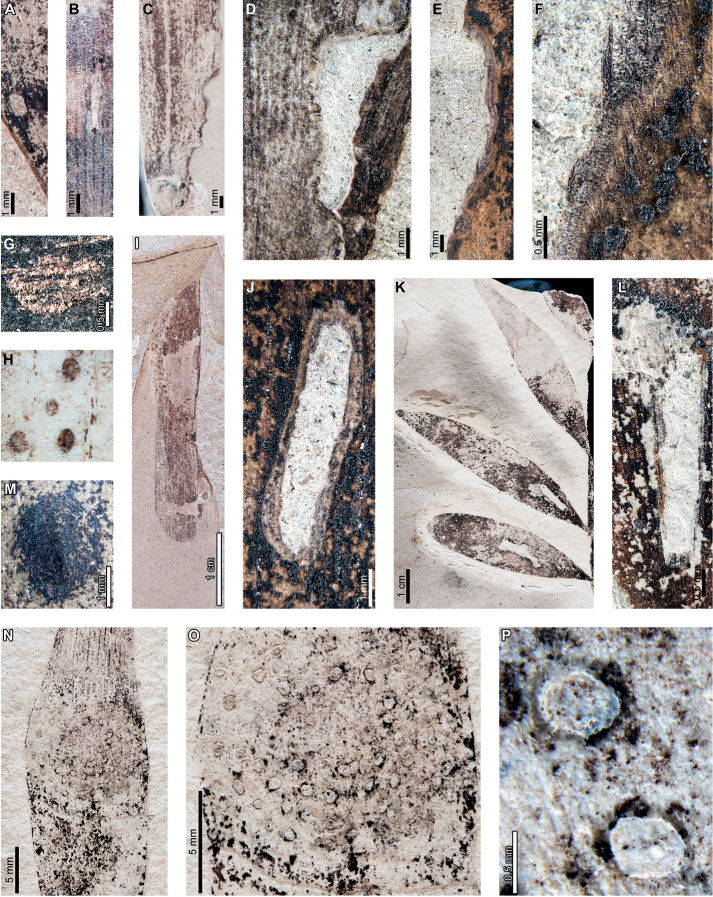
External foliage feeding, leaf mining, and rust fungus damage on *Agathiszamunerae* from Laguna del Hunco **A** oval hole (DT1; MPEF-Pb 6328) **B** parallel-sided slot feeding (DT8; MPEF-Pb 6368) **C** excision into the leaf margin (DT12; MPEF-Pb 6329) **D** adjacent, shallow excisions into the leaf margin (DT12; MPEF-Pb 6311) **E** shallow excision into the leaf margin with veinal stringers (DT12; MPEF-Pb 6361) **F** detail of veinal stringers and reaction tissue in (**E**) **G** small zone of surface feeding (DT29; MPEF-Pb 6356) **H** circular to elliptical piercing and sucking marks (DT46; MPEF-Pb 6303) **I***Frondicuniculumlineacurvum* blotch mine (holotype MPEF-Pb 6336) **J** probable blotch mine with breached epidermal tissue (DT251; MPEF-Pb 6361) **K** two probable blotch mines with breached epidermal tissue (DT251) and a rust fungus (MPEF-Pb 6303) **L** detail of blotch mine in (**K**) **M** dark, circular gall with slight relief (DT32; MPEF-Pb 6346) **N** concentric rings of aecia on a rust fungus spot (MPEF-Pb 6303) **O** detail of rust fungus in (**N**) **P** detail of two aecia in (**N**).

Insect and pathogen damage on early Eocene *A.zamunerae* at LH in the Huitrera Formation includes hole feeding, margin feeding, surface feeding, piercing and sucking, mining, galling, and a rust fungus. External foliage feeding (Fig. 6A–G) includes circular to elliptical holes measuring 0.2–1.1 mm in length and 0.1–0.7 mm in width (DT1; Fig. 6A). The holes are surrounded by thin reaction rims, which measure <0.1 mm wide. A slot-feeding hole (DT8; Fig. 6B) measures 2.8 mm long and 0.3–0.6 mm wide with a 0.1 mm wide reaction rim. The long axis of the hole is parallel to the leaf veins. Shallow, approximately circular excisions into the leaf margins (DT12, Fig. 6C–F) measure 1.0–28.2 mm long and 0.2–2.8 mm deep. Flaps of unconsumed, apparently necrotic tissue measure 0.1–0.5 mm wide, and veinal stringers may be present (Fig. 6F). Polylobate surface feeding zones with reaction rims (DT30; Fig. 6G) measure 1.3–3.0 mm long by 0.2–1.3 mm wide. Reaction rims measure 0.2 mm wide.

Circular to elliptical piercing-and-sucking punctures (DT46; Fig. 6H) are characterized by a black spot or a rim encircling an inner circle of lighter tissue where the puncture was located. These piercing-and-sucking punctures measure 0.11–0.18 mm in minimum and 0.14–0.22 in maximum diameter.

*Agathiszamunerae* is associated with the ichnotaxon *Frondicuniculumlineacurvum*, an oblong blotch mine with smooth, gently curving margins described previously (Donovan et al. 2020). The mines follow the leaf axis, and their long axes are parallel to leaf veins. Frass, including amorphous matter and spherical to hemispherical pellets locally replaced by amber, is deposited mostly along the mine margin when present (Fig. 6I). Putative linear blotch mines with smooth margins and breached epidermal tissue are also found on *A.zamunerae* (DT251; Fig. 6J–L). Epidermal tissue was breached on most specimens, leaving flaps of tissue along the internal margin of the mine, although epidermal tissue is still preserved across the width of some mines (Fig. 6L). Detailed descriptions of these mines, including the ichnotaxomic treatment of *F.lineacurvum*, were provided by Donovan et al. 2020.

A dark, oval gall measures 3.4 mm long by 2.8 mm wide (DT32; Fig. 6M). The long axis of the gall is parallel to the leaf venation. The gall has slight relief above the leaf surface and is characterized by a smooth, carbonized texture.

**Figure 7.**
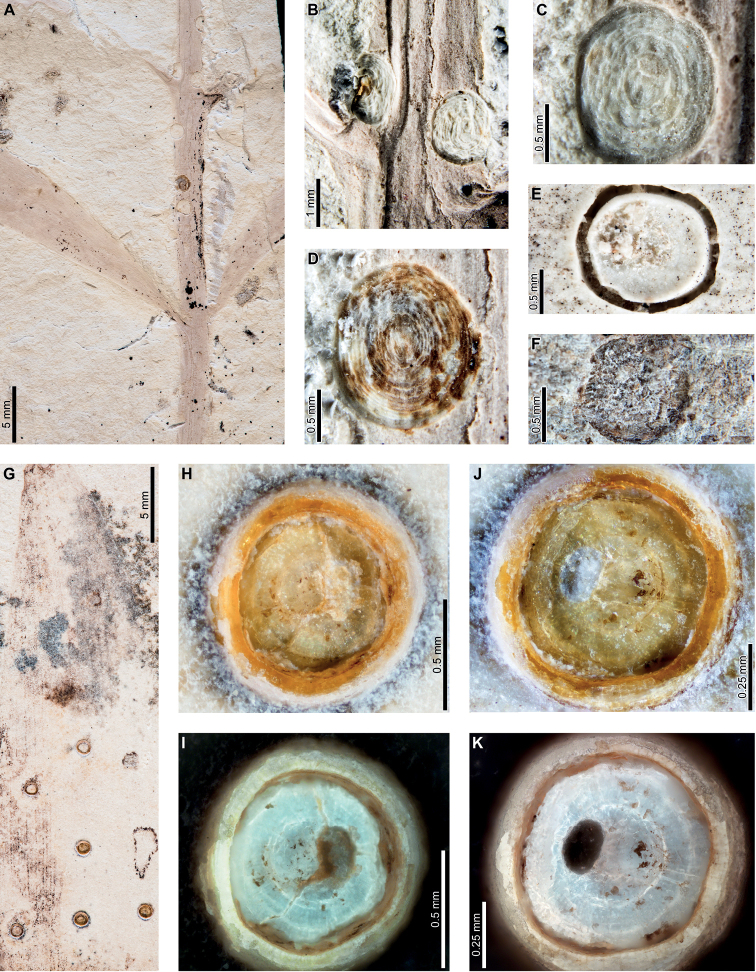
Enigmatic structures, possibly armored scale insect (Diaspididae) covers (DT86) on *Agathiszamunerae* branches (**A–D**) and leaves (**E–K**) from Laguna del Hunco **A** impressions of covers (MPEF-Pb 6307) **B** two covers on a branch in (**A**) **C** detail of cover with concentric growth rings in (**A**) **D** detail of cover with concentric growth rings in (**A**) **E** depression where ventral cover was positioned (MPEF-Pb 6349) **F** depression where cover was probably located (MPEF-Pb 6360) **G** possible scale insect covers (MPEF-Pb 6383) **H** detail of cover in (**G**) **I** cover in (**H**) under epifluorescence showing concentric growth rings **J** detail of cover in (**G**) **K** cover in (**J**) under epifluorescence showing concentric growth rings.

**Figure 8.**
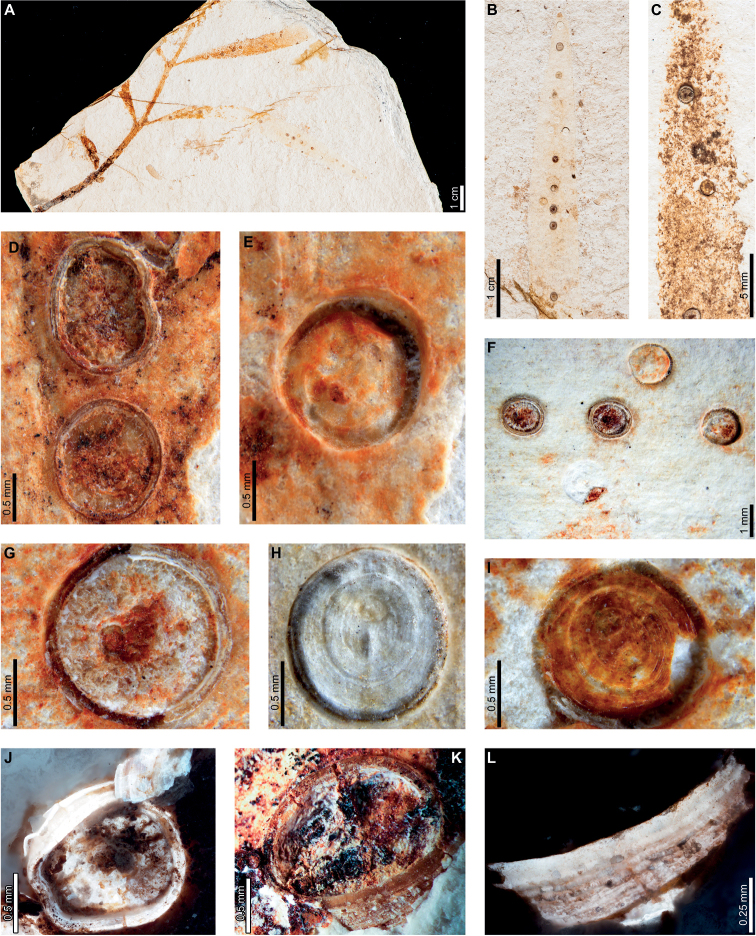
Enigmatic structures, possibly armored scale insect (Diaspididae) covers (DT86) on *Agathiszamunerae* from Laguna del Hunco (MPEF-Pb 6324) **A** covers on leaves and a branch **B** row of covers along the central axis of a leaf **C** four covers **D** dorsal and ventral covers **E** impression of cover **F** five covers **G** ventral cover and poorly preserved dorsal cover **H** impression of dorsal cover with concentric growth rings **I** dorsal cover with concentric growth rings **J** view of ventral cover under epifluorescence **K** protruding ventral cover with horizontal and vertical striations **L** ventral cover with horizontal and vertical striations under epifluorescence.

**Figure 9.**
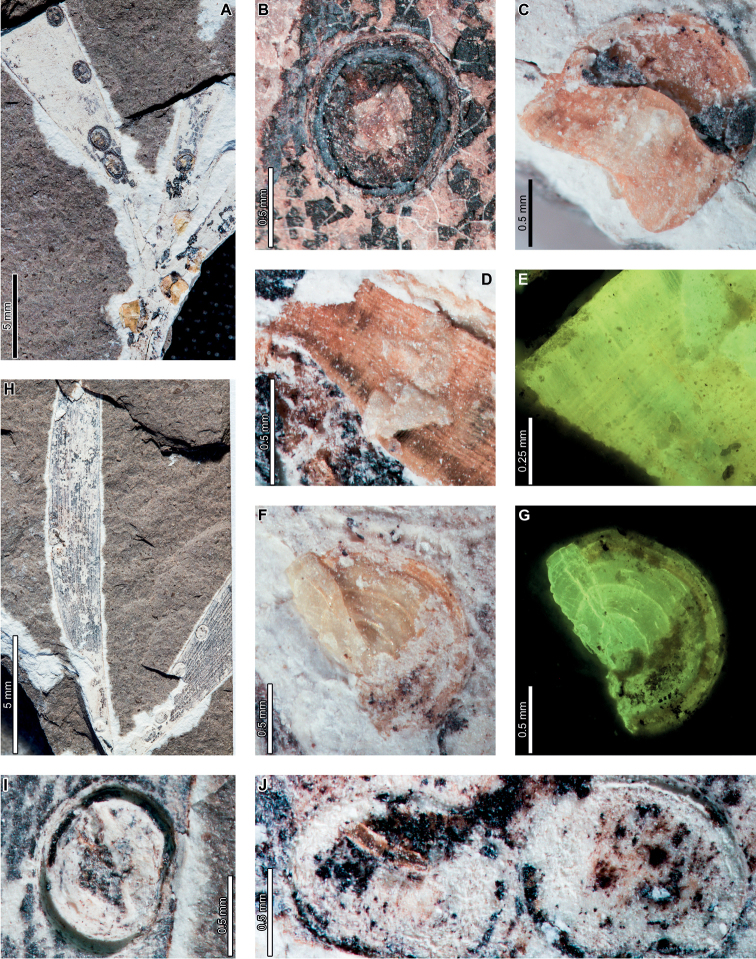
Enigmatic structures, possibly armored scale insect (Diaspididae) covers on *Agathiszamunerae* from Laguna del Hunco (MPEF-Pb 9842) **A** cover impressions and amber casts on leaves and a branch **B** raised ventral cover **C** dorsal cover surrounded by exposed, vertically-oriented ventral cover **D** detail of ventral cover showing horizontal and vertical striations **E** ventral cover in (**D**) under epifluorescence **F** dorsal cover with concentric growth rings **G** dorsal cover in (**F**) **H** counterpart to (**A**) **I** impression of ventral cover in (**H**) **J** impressions of ventral covers in (**H**).

Enigmatic structures, possibly representing female diaspidid covers, are flattened, approximately circular to oval, and measure 0.90–1.63 mm in diameter (DT86; Figs 7–9). In our descriptions, we use the term “covers” to refer to these structures. The dorsal covers are marked by concentric rings (Figs 7B–D, H–K, 8H, I, 9F, G), which may represent cover growth increments during the first and second instar and adult phases. The first instar covers measure 0.31–0.44 mm minimum by 0.29–0.57 mm maximum diameter. The covers constructed by the first instars are marked by an ovoid depression along the edge of the cover, which may indicate the position of the remnant of the first instar exuviae or the stylet fascicle as it penetrated subjacent epidermal and deeper tissues (Figs 7H–K, 8G–I). The ovoid depressions measure 0.22–0.24 mm long by 0.14–0.18 mm wide, with the long axes typically parallel to the maximum diameter of the entire dorsal cover. Material added by second instars increases the diameters of the dorsal covers to 0.58–0.92 mm by 0.62–0.75 mm, and the fully formed adult dorsal covers measure 0.7–1.47 mm by 0.7–1.33 mm. Each dorsal cover is surrounded by a ventral cover (Figs 8J–L, 9C–E), which is deeply embedded in the leaf tissue and measures 0.04–0.15 mm wide. The tops of the ventral covers typically protrude above the leaf surface and the dorsal covers. Although the entire heights of the ventral covers are not usually visible, measured heights are 0.55–0.75 mm. Some scales are only represented by pits (Fig. 8E) or depressed rims where the ventral cover was originally positioned but subsequently detached (Figs 7E, 9I, J).

A probable rust fungus (Fig. 6K, N–P) developed as a gall on which nearly concentric rings of circular to oval aecia (aeciospore-producing structures) are embedded. The aecia are 0.45–1.28 mm in diameter and have slight relief relative to the leaf surface (Fig. 6P). A depressed rim, 0.10–0.25 mm wide, surrounds each aecium, suggesting that the aecia were deep-set in the host tissue and/or that the aecia were cupulate. The circular gall in which the aecia are embedded is delimited by a depressed rim (0.3 mm wide), most clearly visible on the basal side of the gall as oriented in Fig. 6P. Leaf veins within the spot are distorted, possibly caused by thickened tissue growth. The gall measures 13.2 mm in the direction parallel to the leaf veins, and it spans the width of the leaf (15.6 mm).

### ﻿Middle Eocene, Río Pichileufú

**Figure 10.**
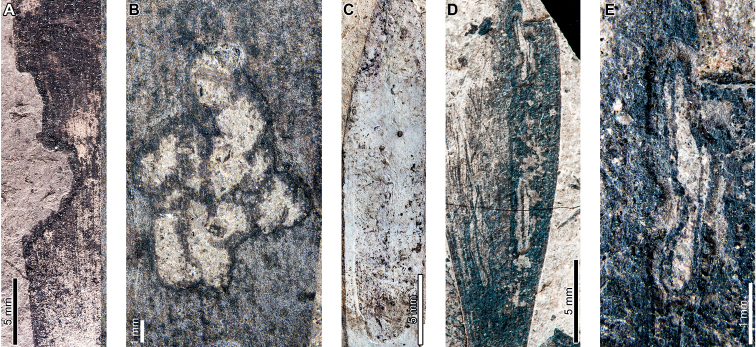
External foliage feeding and leaf mines on *Agathiszamunerae* leaf from Río Pichileufú **A** arcuate excisions into the leaf margin (DT12; BAR 5002-20) **B** zone of skeletonized tissue with reaction rims (DT17; USNM 545229) **C***Frondicuniculumlineacurvum* blotch mine (paratype USNM 545226) **D** probable blotch mines with breached epidermal tissue (DT251; USNM 545227) **E** detail of blotch mine in (**D**).

Insect damage on early middle Eocene *A.zamunerae* at RP in La Huitrera Formation includes margin feeding, skeletonization, and mining. External foliage feeding (Fig. 10A, B) includes an excision into the leaf margin composed of two, adjacent, approximately circular feeding areas (DT12; Fig. 10A) that measure 13 mm across and are incised 1.8 and 3.7 mm, respectively. Ragged reaction tissue with visible vein stringers measures 0.6–0.8 mm wide. A patch of skeletonized tissue composed of multiple, closely spaced, holes with some leaf veins intact is surrounded by a dark reaction rim (DT17; Fig. 10B) measuring 0.3–0.5 mm wide.

The ichnotaxon *Frondicuniculumlineacurvum*, an elongate-ellipsoidal blotch mine with smooth margins, occurs on *A.zamunerae* at both RP and LH, as previously reported (Donovan et al. 2020). The best-preserved example occupies approximately 85% of a leaf surface (Fig. 10C). Putative oblong blotch mines with flaps of unconsumed tissues along the rims of the mines are also found on *A.zamunerae* at RP (DT251; Fig. 10D, E). Detailed descriptions of these mines were provided by Donovan et al. 2020.

**Figure 11.**
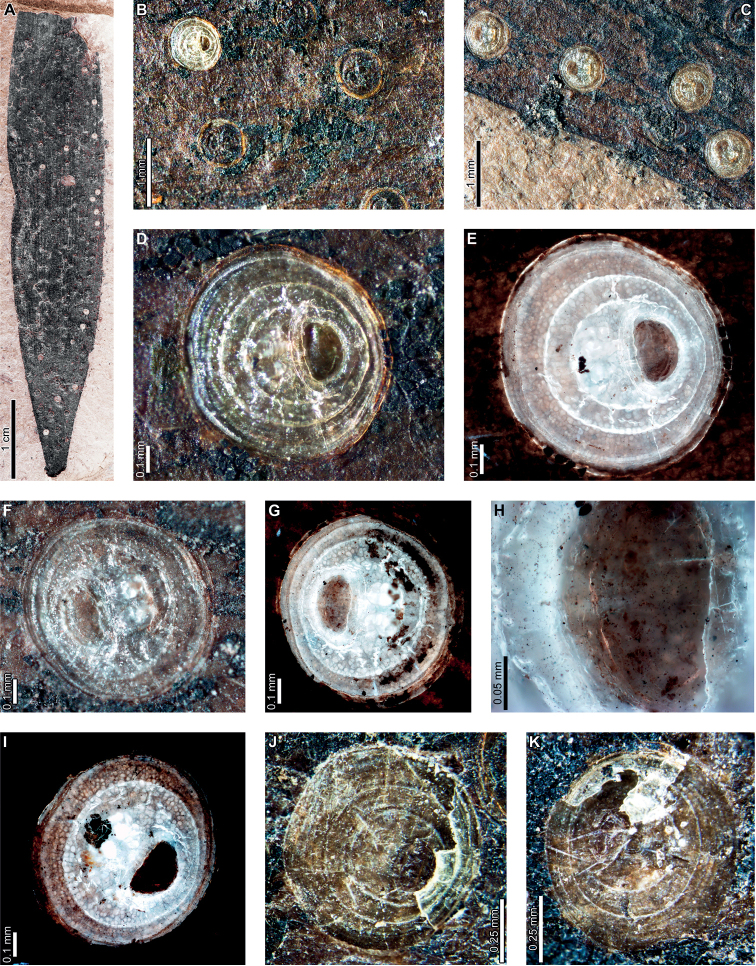
Enigmatic structures, possibly armored scale insect (Diaspididae) covers (DT86) on an *Agathiszamunerae* leaf from Río Pichileufú (USNM 545228) **A** leaf with covers **B** dorsal covers **C** dorsal covers **D** dorsal cover with concentric growth rings **E** cover in (**E**) under epifluorescence **F** dorsal cover with concentric growth rings **G** cover in (**F**) under epifluorescence **H** detail of rim surrounding semicircular impression in (**G**) **I** dorsal cover under epifluorescence showing concentric growth rings **J** dorsal cover with concentric growth rings **K** dorsal cover with concentric growth rings.

**Figure 12.**
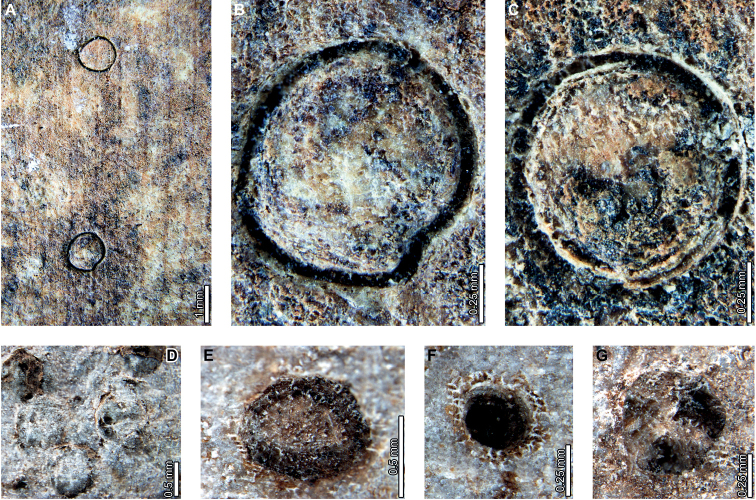
Enigmatic structures, possibly armored scale insect (Diaspididae) cover impressions (DT86) on *Agathiszamunerae* from Río Pichileufú **A** depressions where ventral covers were positioned (USNM 545223) **B** detail of lower depression in (**A**) **C** rim where ventral cover was positioned (USNM 545223) **D** pits where scale insects were possibly positioned (USNM 545226) **E** pit where scale insect was possibly located (USNM 545226) **F** pit surrounded by reaction tissue where scale insect was possibly positioned (USNM 545226) **G** Pit where scale insect was possibly located (USNM 545226).

Enigmatic structures resembling female diaspidid covers (Figs 11, 12) are similar to those found at LH. Dorsal covers measure 0.70–1.08 mm in diameter and are marked by two concentric rings (0.40 by 0.38 mm and 0.53–0.55 by 0.55–0.57 mm wide, respectively), possibly representing instar growth increments (DT86; Fig. 11). The first instar covers are usually associated with an oval or semicircular depression (0.22–0.23 mm long by 0.15–0.16 mm wide; Fig. 11D–I) surrounded by a raised rim or flange (0.02–0.06 mm wide; Fig. 11H). Dorsal covers are marked by small circular to ellipsoidal shapes visible under epifluorescence (Fig. 11E, G, I), which correspond to bumps on the surface. The bumps measure 0.01–0.02 mm in diameter and decrease in size moving from the first instar through the adult portions of the scales. The first instar covers appear to be thicker than the material added by second instars and adults (Fig. 11E, G, I), probably attributable to the presence of exuviae. A deeply-set ventral cover (0.02–0.06 mm wide) surrounds each dorsal cover (Fig. 12A–C). Ovoid pits may represent where covers were originally positioned (Fig. 12D–G).

### ﻿Extant *Agathis* herbivory

Relatively few herbivorous insect associations with extant *Agathis* have been documented in the literature, as summarized by Donovan et al. (2020). Here, we provide brief descriptions of insect and fungal damage that we observed on *Agathis* herbarium specimens. Although not intended to be a comprehensive survey, the damage is representative of the diversity we found while looking for analogs to the fossil damage on thousands of herbarium sheets in several major herbaria. Much of this extant damage diversity that resembles the fossils is from unknown culprits, representing a major opportunity to discover living insect diversity and evolutionary history based on fossil analogs. Some of the leaf mines were previously illustrated by Donovan et al. (2020) but are re-illustrated and briefly mentioned in the text here for completeness. Extant *Agathis* species delimitations and range information follows (Farjon 2010). See the following section for comparisons of the fossil and extant damage.

**Figure 13.**
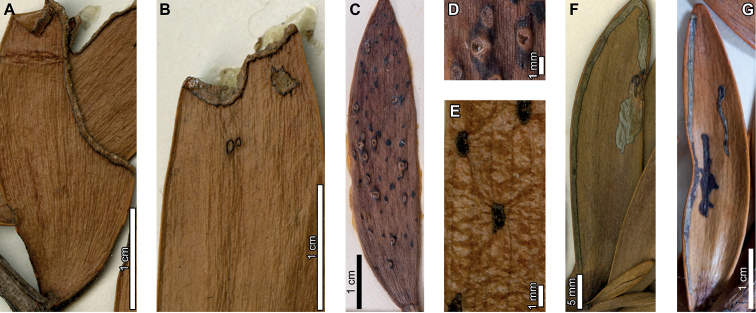
Insect damage on *Agathisaustralis* from New Zealand **A** excision into the leaf margin (Capt. Wilkes, U.S.N., 1838-42 (GH)) **B** excision through the leaf apex (Capt. Wilkes, U.S.N., 1838-42 (GH)) **C** blister galls with exit holes (K 0000230) **D** detail of blister galls in (**C**) **E** black, ellipsoidal galls (E.H. Wilson, February 2, 1921 (A)) **F***Parectopaleucocyma* (Gracillariidae) moth mine (Capt. Wilkes, U.S.N., 1838-42 (GH)) **G***Parectopaleucocyma* mines (K 000553313).

On *Agathisaustralis* (Fig. 13; from the North Island of New Zealand), we observed external foliage feeding, including arcuate margin feeding (DT12; Fig. 13A) and removal of the leaf apex (DT13; Fig. 13B). A flap of dead tissue bordered by a black reaction rim forms at the sites of external foliage feeding damage (Fig. 13A, B). Galls are black, circular to ellipsoidal, with minor relief compared to the leaf surface (Fig. 13C–E). Most galls are single-chambered, but some are conjoined or are multi-chambered with exit holes. Galls were found on the adaxial leaf surfaces, and the long axes of the galls tend to be oriented parallel to leaf veins. Finally, *Parectopaleucocyma* (Lepidoptera, Gracillariidae) (Wise 1962) mines are common, typically characterized by an initial blotch phase transitioning into a serpentine trail. The mines follow the leaf margin and end in a gall near the petiole (Fig. 13F, G).

**Figure 14.**
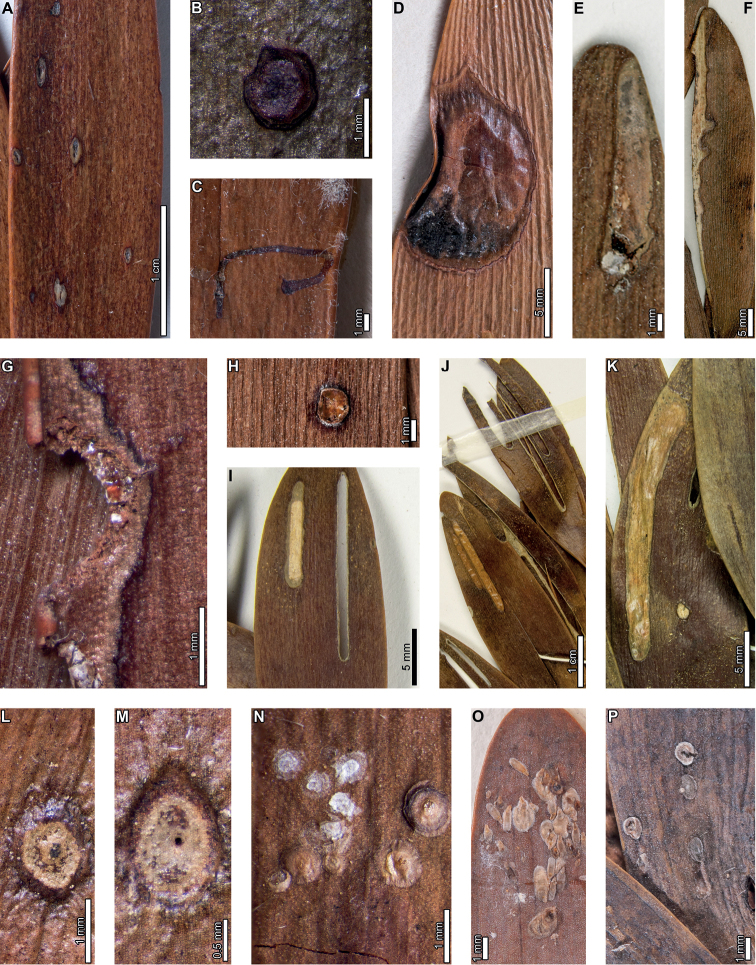
Insect and pathogen damage on *Agathislanceolata* (**A–E**), *A.montana* (**F**), *A.moorei* (**G–K**), *A.ovata* (**L–P**) from New Caledonia **A** ellipsoidal blister galls with exit holes (K 000553125) **B** black columnar gall (E 00119757) **C** Serpentine mine (K 000553127) **D** teardrop-shaped blotch damage (K 0000305) **E** linear blotch damage (GH 2372 B) **F** elongate mine along the leaf margin (A 8571) **G** excision into the leaf margin surrounded by reaction tissue and exuding resin (E 00106192) **H** circular gall (K 0000357) **I** linear blotch mines with breached epidermal tissue on the right mine (K 000553352) **J** blotch mines (K 000553352) **K** blotch mine (K 000553352) **L, M** galls with thickened margins surrounding epidermal tissue with circular exit holes (E 00399687) **N***Chrysomphalusaonidum* (Diaspididae) scale insects (E 00036880) **O** diaspidid scale insect covers (K 0000291) **P** diaspidid scale insect covers (K 00053124).

*Agathislanceolata* (New Caledonia; Fig. 14A–E) is associated with external foliage feeding, including semicircular excisions into the leaf margin (DT12) and removal of the leaf apex (DT13). Galls on *A.lanceolata* include ellipsoidal blisters oriented parallel to leaf veins (Fig. 14A). Houard (1914, 1922) briefly described ellipsoidal blister galls on the upper surfaces of *A.lanceolata* leaves. The galls were not illustrated, but he may have been referring to galls similar to those in Fig. 14A. A dark, circular gall with a flattened top and a central indentation was found on a cultivated tree in New Caledonia (Fig. 14B). A curved serpentine mine with smooth margins is packed with frass (Fig. 14C). Two blotches of indeterminate origins, possibly induced by pathogens (fungi, viruses, or bacteria), were found on *A.lanceolata*. First, a teardrop shaped blotch with raised epidermal tissue that measures 15.1 mm long by 7.7–8.7 mm in width (Fig. 14D) has an acuminate upper margin and is surrounded by a wrinkled reaction rim 0.3–0.4 mm wide. The second blotch is oblong and is positioned along the leaf edge (Fig. 14E). The blotch is 14.4 mm long by 1.4–3.1 mm wide.

On *Agathismontana* (New Caledonia), we observed an elongate mine following the margin of a single leaf (Fig. 14F). The mine measures 59.7 mm long by 0.2–4.3 mm wide and is surrounded by a 0.1 mm wide reaction rim. The mine begins near the base of the leaf and follows the leaf margin, terminating at the leaf apex. The mine gradually widens as the miner consumed leaf tissue, except for two expanded protrusions, and spans the width of the leaf at the apex. The lateral margins of the mine are smooth, and linear to gently undulous frass appears to be composed of spheroidal pellets.

*Agathismoorei* (New Caledonia; Fig. 14G–K) is associated with shallow, arcuate margin-feeding excisions (DT12; Fig. 14G) flanked by lighter colored dead tissue and dark reaction rims. The wounded edges may exude resin. Galls are circular with minor relief and a raised black rim (Fig. 14H). Full-depth blotch mines are falcate or linear and smooth with parallel margins (Fig. 14I–K). Epidermal tissue was weathered away in some specimens (Fig. 14I, J).

*Agathisovata* (New Caledonia; Fig. 14L–P) is associated with ellipsoidal to ovate galls with a dark, thickened rim (Fig. 14L, M). The tops of the galls are flat and consist of epidermal tissue with distinct files of cellular proliferations and a circular exit hole. Diaspidids, including *Chrysomphalusaonidum* (Fig. 14N) and unidentified species (Fig. 14O, P) also occur on this species.

**Figure 15.**
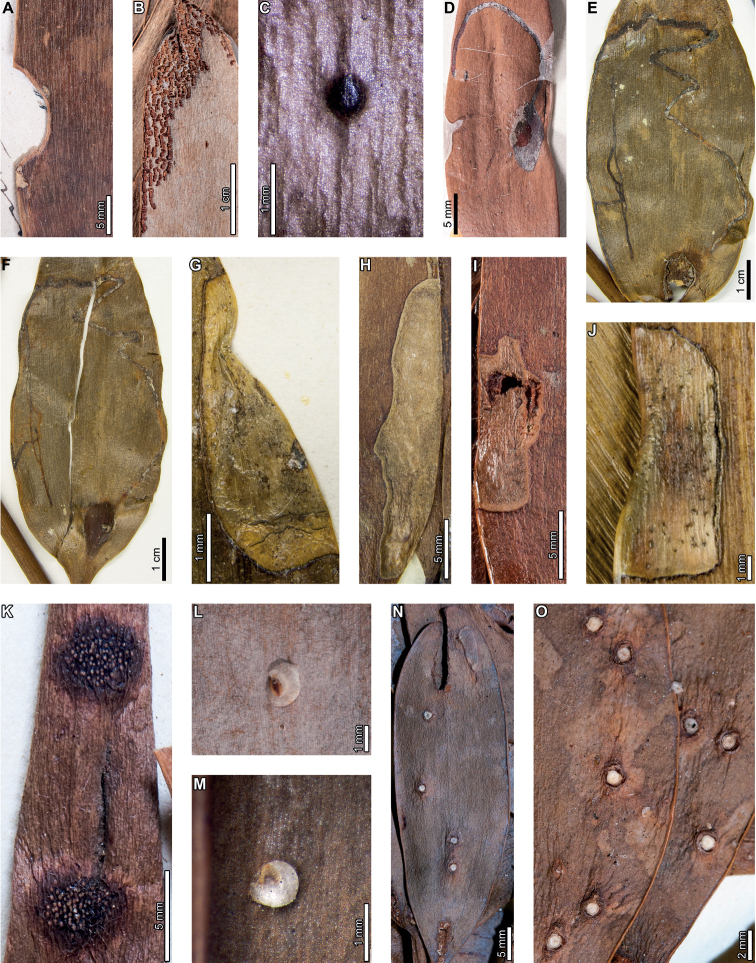
Insect and fungal damage on *Agathismacrophylla* (**A–M**) **A** semicircular excision into the leaf margin (K 0000265) **B** adjacent rows of surface feeding (Fiji, K B40) **C** round, black gall (New Caledonia, E 00131862) **D** serpentine mine with elliptical terminal chamber (Fiji, E 0000340) **E** serpentine mine terminating in a gall (Fiji, K 0000346) **F** serpentine mine terminating in a gall (Fiji, K 0000346) **G** possible blotch mine along leaf margin (Fiji, K 0000322) **H** elongate blotch mine (Fiji, K 0000327) **I** blotch mine along the leaf margin (Fiji, K 0000327) **J** fungal blotch along the leaf margin (Fiji, K 16421) **K** Kauri rust (*Araucariomycesfragiformis*) (Vanuatu, S.F. Kajewski 282 (K)) **L** armored scale insect (Diaspididae) (Fiji, K 350) **M** diaspidid scale insect (Fiji, E 00127892) **N, O** pit gall-inducing scale insects (Fiji, GH 01153259).

*Agathismacrophylla* (Fiji, Vanuatu, and the Solomon Islands; Fig. 15A–O) is associated with semicircular excisions into the leaf margin (DT12; Fig. 15A) and feeding through the leaf apex (DT13) with flaps of dead tissue bordered by a rim of darkened tissue. Surface feeding consists of thin, fairly linear files of damaged tissue (Fig. 15B). Galls include hemispherical, black protrusions from the adaxial surface (Fig. 15C) of leaves and small, circular blister galls with central exit holes. Serpentine mines are curved and end in an elliptical terminal chamber (Fig. 15D) or travel in a zigzag pattern and terminate with a gall at the leaf base (Fig. 15E, F). The terminal gall is similar to mines made by *Parectopaleucocyma* on *Agathisaustralis* in New Zealand, although *P.leucocyma* mines have an initial blotch phase (Wise 1962) and do not mine in a zigzag pattern. Elongate, oblong to elliptical blotch mines are positioned along leaf margins with their long axes parallel to leaf veins (Fig. 15G–I). Diaspidid scale insects cause piercing-and-sucking damage, leaving a discolored orange mark where the scale cover was located (Fig. 15L, M). Pit-gall-inducing diaspidids are also associated with leaves of *A.macrophylla* (Fig. 15N, O). The only other gall-inducing diaspidid that has been documented on conifers is *Leucaspispodocarpi*, which makes leaf margin rolls on *Prumnopitystaxifolia* (Podocarpaceae) in New Zealand. *Leucaspispodocarpi* is also associated with *Podocarpuscunninghamii* Colenso and *Podocarpustotara* G.Benn ex D.Don (Podocarpaceae), but it does not induce galls on these species (Henderson and Martin 2006). A blotch, probably fungal-induced, occurs along the leaf margin (Fig. 15J). Kauri rust (*Aecidiumfragiforme*) fungal galls are also common (Fig. 15K).

**Figure 16.**
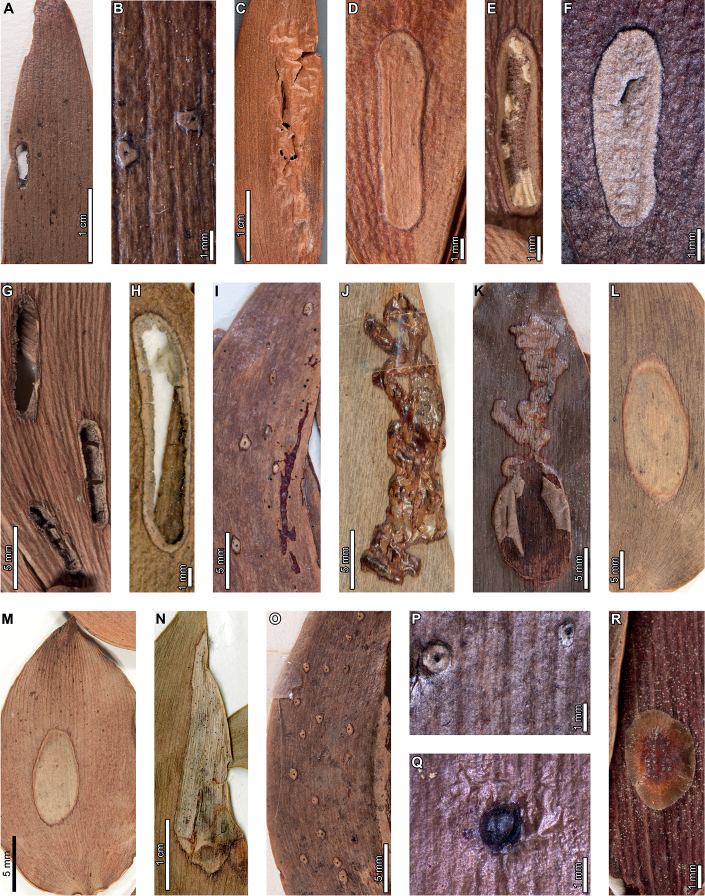
Insect damage on *Agathisatropurpurea* (**A–C**), *A.microstachya* (**D–F**), and *A.robusta* (Fig. I–R) **A** slot feeding (Australia, K 000553290) **B** blister galls with circular exit holes (Australia, K 000553290) **C** elongate blotch mine (Mount Bartle Frere leaf litter, Queensland, Australia) **D** ellipsoidal blotch mine (Australia, K 0000199) **E** blotch mine lacking epidermal tissue (Australia, K 0000199) **F** ellipsoidal blotch mine (Australia, E 00210640) **G** ellipsoidal blotch mines (Australia, K 111455) **H** blotch mine (Queensland, Australia; A.K. Irvine 00417 (A)) **I** ellipsoidal blister galls with exit holes (Queensland, Australia, K 000553277) **J** serpentine mine (Australia, CANB 590252) **K** serpentine mine with ellipsoidal terminal chamber (Australia, K 000553286) **L** ovate blotch, probable pathogen damage (Australia, NSW 381650) **M** ovate blotch, probable pathogen damage (Australia, NSW 381650) **N** elongate fungal blotch (Queensland, Australia, A 01153261) **O** ellipsoidal blister galls with exit holes (New Guinea, K 000553264) **P** circular blister galls with exit holes (New Guinea, E 00127882) **Q** globose, black gall (New Guinea, E 00127880) **R** coccid scale insect (New Guinea, K 000553265).

*Agathisatropurpurea* (Queensland, Australia; Fig. 16A–C) is associated with slot feeding (DT8; Fig. 16A) characterized by a thin rim of necrotic tissue along the inside of the hole. Ovoid to elliptical and polylobate blister galls are positioned on the adaxial sides of leaves and are marked by centrally located circular exit holes (Fig. 16B). An oblong blotch mine with smooth, gently curving margins is positioned near the leaf margin (Fig. 16C).

*Agathismicrostachya* (Queensland, Australia; Fig. 16D–F) is associated with elongate-ellipsoidal blotch mines that have smooth margins positioned with their long axes parallel to the leaf veins. In one specimen (Fig. 16E), the epidermal tissue was removed due to weathering, revealing an abraded inner texture caused by insect feeding.

*Agathisrobusta* occurs in both Queensland, Australia (Fig. 16G–K) and New Guinea (Fig. 16L–O). In the Australian material, we found semicircular excisions along the leaf margin (DT12) and apex feeding (DT13). Probable blotch mines with breached epidermal tissue have a slot-like appearance. Ellipsoidal blister galls are characterized by a central exit hole and are surrounded by black rims (Fig. 16I). Serpentine mines are tightly wound (Fig. 16J, K) and some end in an elliptical terminal chamber (Fig. 16K). Ovate blotches caused by pathogens are positioned near the central axes of leaves (Fig. 16L, M) and measure 17.7–20.5 mm long by 8.6–8.9 mm at their widest diameter. The margins of the blotches are minutely ragged and surrounded by 0.1–0.2 mm wide reaction rims. Elongate fungal blotches occur along the leaf margin (Fig. 16N). In New Guinea specimens, *A.robusta* is associated with small circular holes (DT2). Blister galls on *A.robusta* in New Guinea (Fig. 16O, P) are indistinguishable from those found on Australian members of the species (Fig. 16I). Globose, black galls (Fig. 16Q) and a single coccid scale insect (Fig. 16R) were also found on the leaf surface.

**Figure 17.**
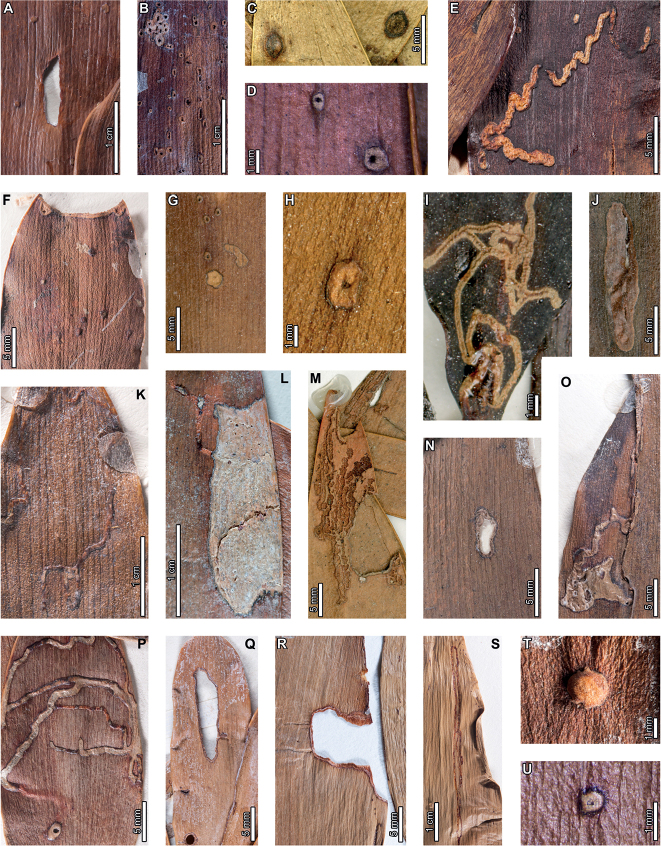
Insect damage on *Agathislabillardieri* (**A–E**) and *A.dammara* (**F–U**) **A** slot feeding (New Guinea, K 0000171) **B** densely-packed blister galls (New Guinea, K 0000161) **C** elliptical galls composed of brown, thickened tissue (New Guinea, A 63040) **D** circular blister galls with exit holes (New Guinea, E 00036878) **E** sinusoidal serpentine mine (New Guinea, SING NIFS bb.30358) **F** feeding along the leaf apex (Maluku Islands, K 000553215) **G** polylobate blister galls with exit holes (Maluku Islands, A 106) **H** blister gall with circular exit hole (Maluku Islands, A 106) **I** serpentine mine with thin frass trail (Maluku Islands, A 121) **J** ellipsoidal blotch mine (Maluku Islands, A 121) **K** serpentine mine (Maluku Islands, K 0000041) **L** serpentine mine ending in blotch (Maluku Islands, K 0000078) **M** elongate rows of parallel surface feeding (Brunei, A 4330) **N** slot feeding (Sulawesi, Indonesia, K 0000067) **O** serpentine mine terminating in a blotch (Sulawesi, Indonesia, K 0000067) **P** serpentine mine with oval terminal chamber (Sulawesi, Indonesia, K 516) **Q** slot feeding (Philippines, K 0000137) **R** excision into the leaf margin that expands towards the center of a leaf (Philippines, K 3091) **S** linear serpentine mine (Philippines, K 3091) **T** globose woody gall (Philippines, K 000553230) **U** blister gall with central exit hole and darkened rim (Java, Indonesia, E 00420594).

*Agathislabillardieri* (New Guinea; Fig. 17A–E) is associated with external foliage feeding, including small circular holes (DT2), slot feeding (DT8; Fig. 17A), semicircular excisions into the leaf margin (DT12), apex feeding (DT13), and linear traces of surface feeding that parallel the leaf veins. Galls include densely packed blisters with single or multiple chambers (Fig. 17B, D) and elliptical galls composed of brown, thickened tissue (Fig. 17C). Sinusoidal serpentine mines also occur on *A.labillardieri* (Fig. 17E).

**Figure 18.**
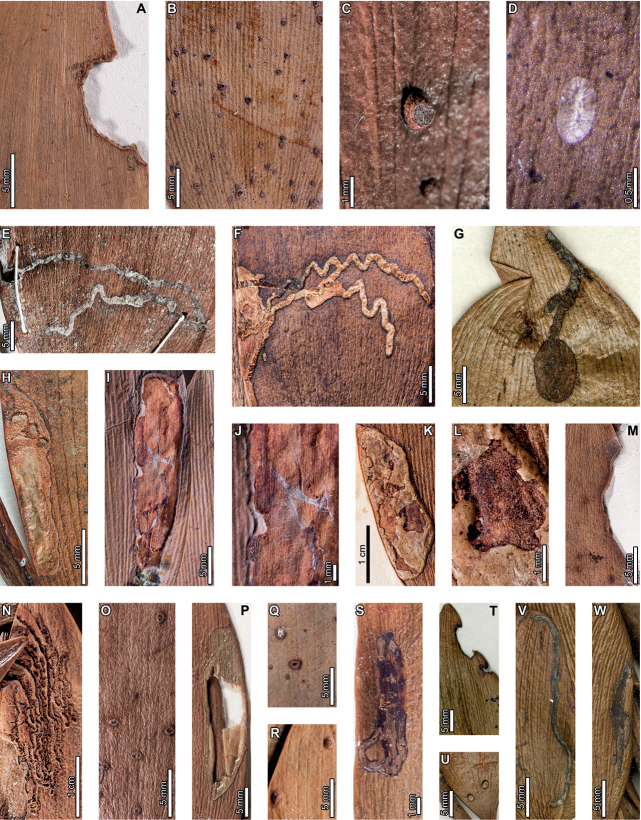
Insect damage on *Agathisborneensis* (**A–L**), *A.lenticula* (**M–P**), *A.kinabaluensis* (**Q–S**), *A.flavescens* (**T–W**) **A** arcuate margin feeding (Sarawak, Malaysia, K 17672) **B** ellipsoidal to polylobate blister galls with exit holes (Kalimantan, Indonesia, A B1468) **C** hardened, cylindrical galls with flat tops (Brunei, K 000553181) **D** coccid scale insect (Kalimantan, Indonesia, E 00032242) **E** serpentine mine (Penang Island, Malaysia, K 000553157) **F** sinusoidal serpentine mine (Penang Island, Malaysia, SING 90799) **G** serpentine mine with terminal chamber (Sulawesi, Indonesia, A 4109) **H** elongate blotch mine (Sarawak, Malaysia, K 000553188) **I** blotch mine with silk (Brunei, K 000553179) **J** detail of silk in mine in (**I**) **K** blotch mine containing frass (Brunei, SING 91231) **L** close-up of frass in blotch mine in (**K**) **M** arcuate margin feeding (Sabah, Malaysia, K 000553185) **N** adjacent rows of surface feeding (Sabah, Malaysia, K 000553187) **O** circular to polylobate galls with flat tops and exit holes (Sabah, Malaysia, K 000553185) **P** elongate blotch mine along the leaf margin (Sabah, Malaysia, K 000553187) **Q** circular to ellipsoidal galls with exit holes (Sabah, Malaysia, K 000553243) **R** circular, raised galls (Sabah, Malaysia, K 000553243) **S** tightly overlapping serpentine mine (Sabah, Malaysia, K P644) **T** shallow excisions into the leaf margin **U** blister galls surrounded by a thin black rim (Malaysia, A P544) **V** serpentine mines packed with frass (Malaysia, A P542) **W** overlapping serpentine mine (Malaysia, A P542).

On *Agathisdammara* (eastern Malesia; Fig. 17F–U), external foliage feeding includes circular (DT2) and polylobate (DT5) holes, slot feeding (DT8; Fig. 17N, Q), arcuate excisions (DT12), feeding through the leaf apex (DT13; Fig. 17F), and trenched excisions into the leaf margin that expand towards the center of the leaf (DT15; Fig. 17R). Elongate, squiggly rows of surface feeding run parallel to each other (Fig. 17M). Circular to polylobate blister galls with central circular exit holes (Fig. 17G, H, U) and a globose woody gall (Fig. 17T) are found on the adaxial surfaces of leaves. Various serpentine mine morphologies occur on *A.dammara*, including overlapping (Fig. 17I), forking (Fig. 17K), or linear trails (Fig. 17S) that end in an elongate blotch (Fig. 17L), polylobate terminal chamber (Fig. 17O), or a gall (Fig. 17P). Elongate-ellipsoidal blotch mines also occur (Fig. 18J).

*Agathisborneensis* (Borneo to Sumatra; Fig. 18A–L) is associated with external foliage feeding, including arcuate margin feeding (DT12; Fig. 18A) and apex feeding (DT13) with a thin band of thickened reaction tissue. Galls on the adaxial leaf surface include ellipsoidal to polylobate blister-like bumps with circular exit holes (Fig. 18B) and hardened, cylindrical protrusions with flat tops (Fig. 18C). A coccid scale insect or psyllid (Fig. 18D) was found associated with *A.borneensis*. Mines include winding or overlapping serpentine trails (Fig. 18E, F), serpentine mines transitioning into an oval terminal chamber (Fig. 18G), and oblong to elliptical blotch mines (Fig. 18H–L). The presence of silk in one of the blotch mines suggests that it was created by a lepidopteran caterpillar (Fig. 18I, J).

On *Agathislenticula* (Sabah, Malaysian Borneo; Fig. 18M–P), external foliage feeding includes arcuate excisions into the leaf margin (DT12; Fig. 18M) and curved, adjacent rows of surface feeding (Fig. 18N). Galls are circular to polylobate with flat tops, consist of one or two chambers, and have circular exit holes (Fig. 18O). A possible elongate, elliptical blotch mine or path of pathogen damage occurs along the leaf margin (Fig. 18P) measures 30.7 mm long by 1.0–4.7 mm wide. The miner targeted upper leaf tissue, leaving a thin layer of epidermal tissue intact. The mine has smooth margins, and a 0.1 mm wide enveloping reaction rim. The specimen was damaged, and much of the inner tissue was lost. No evidence of frass is present in the mine.

*Agathiskinabaluensis* (Mt. Kinabalu, Sabah, and Mt. Murud, Sarawak, Malaysian Borneo; Fig. 18Q–S) is associated with galls characterized by hemispherical eminences (Fig. 18Q, R), typically with their long axes oriented parallel to the leaf veins. Some galls are surrounded by a black rim and many have a central circular to elliptical exit hole. The galls appear to be single-chambered. Serpentine mines are characterized by an overlapping path filled with frass in some portions (Fig. 18S).

*Agathisflavescens*, restricted to two mountains in Peninsular Malaysia (Fig. 18T–W), is associated with shallow excisions into leaf margins with black reaction rims (DT12; Fig. 18T). Blister galls surrounded by a thin, black rim and associated with a central, circular exit hole (Fig. 18U) occur on the adaxial surfaces of leaves. Serpentine mines are characterized by densely packed frass, slightly increasing width, and either a curvilinear (Fig. 18V) or overlapping trajectory (Fig. 18W).

## ﻿Comparison of insect damage on fossil and living *Agathis*

Both fossil and extant *Agathis* species are associated with similar, diverse suites of insect and pathogen damage, including external foliage feeding, piercing and sucking, mining, and galling damage. Below, we provide comparisons of damage on *Agathis* across space and time.

### ﻿External foliage feeding

Overall, similar external-feeding damage is found at all four Cretaceous–middle Eocene fossil sites (Table 1). Small, circular holes (DT1) are associated with *Agathis* leaves during the Late Cretaceous (Lef; Fig. 1A), early Paleocene (PL2; Fig. 2A, B), and early Eocene (LH; Fig. 6A). Arcuate excisions into the leaf margins (DT12) occur at Lef (Fig. 1B), PL2 (Fig. 2C, D), LH (Fig. 6C–F), and middle Eocene RP (Fig. 10A); and zones of surface abrasions are found at Lef (Fig. 1C), PL2 (Fig. 2E, F), and LH (Fig. 6G). External foliage feeding damage is made by insects with mandibulate, chewing mouthparts across orders such as Lepidoptera, Coleoptera, and Orthoptera, many of which can produce comparable damage types (Carvalho et al. 2014). Although the presence of similar external foliage feeding damage on *Agathis* at multiple fossil localities does not necessarily suggest that related insects inflicted the damage, the diversity of damage at all fossil sites suggests that *Agathis* species hosted an array of externally feeding herbivores, similar to extant members of the genus (Donovan et al. 2020). Previously documented, extant, external foliage-feeding insects on *Agathis* include chrysomelid and curculionid beetles (Ecroyd 1982), geometrid and tortricid moths (Ecroyd 1982; Nuttall 1983), and phasmids (Plant-SyNZ database 2017) on *Agathisaustralis* in New Zealand. In addition, we found numerous examples of external foliage feeding on herbarium specimens throughout the range of the genus (Figs 13A, B, 14G, 15A, B, 16A, 17A, F, M, N, Q, R, 18A, M, N, T).

**Table 1.** Presence and absence of insect damage types on fossil *Agathis* from Maastrichtian Lefipán localities (Lef), Danian Palacio de los Loros 2 (PL2), early Eocene Laguna del Hunco (LH), middle Eocene Río Pichileufú (RP), and extant *Agathis* from Australasia and Southeast Asia (Donovan et al. 2020).

Functional feeding group	DT	Lef	PL2	LH	RP	Extant analog
*Hole feeding*						
Circular, <1 mm diam.	1	x	x	x		x
Circular, 1–5 mm diam.	2		x			x
Parallel sided slots	8			x		x
*Margin feeding*						
Arcuate excision	12	x	x	x	x	x
*Skeletonization*						
Interveinal tissue removed, reaction rim	17				x	
*Surface feeding*						
Surface abrasion, weak reaction rim	29	x	x	x		x
Polylobate abrasion, strong reaction rim	30			x	x	
*Piercing and sucking*						
Circular punctures, <2 mm diam.	46	x	x	x		x
Scale cover, concentric growth rings	86		x	x	x	x
*Oviposition*						
Ovate scar, prominent reaction rim	101	x				
*Mining*						
Elongate-ellipsoidal blotch, smooth margins	88	x		x	x	x
Serpentine mine, follows parallel veins	139	x				
Linear blotch, breached epidermal tissue	251			x	x	x
Elongate-ellipsoidal blotch, wavy margins	421		x			
*Galling*						
Unhardened central chamber, thickened outer rim	11		x			x
Nondiagnostic, dark, circular	32	x		x		x
Epidermal center, hardened outer walls	115		x			x
Columnar gall	116		x			
*Pathogen*						
Circular epiphyllous rust fungus with concentric aecia	66			x		x

An earlier version of Table 1 was published in Donovan et al. (2020).

### ﻿Piercing and Sucking (fluid feeding)

We found similar circular piercing and sucking marks (DT46) in fossil assemblages spanning the latest Cretaceous to early Eocene (Lef – Fig. 1D; PL2 – Fig. 2G–J; and LH – Fig. 6H), suggesting long-term associations of *Agathis* with hemipteran insects. Evidence for piercing and sucking is common on angiosperms from Lef, PL2 (Donovan et al. 2017), and LH (Wilf et al. 2005a), and on the conifer *Retrophyllum* (Podocarpaceae) from Lef and LH (Wilf et al. 2017). On extant *Agathis*, all known hemipteran associations are scale insects (Figs 14N–P, 15L–N, 16R; Cohic 1958; Williams 1985; Brun and Chazeau 1986; Cox 1987; Williams and Watson 1990; Ben-Dov 1994; Mille et al. 2016), although undocumented associations involving other hemipteran and thysanopteran groups are probable. In addition, kauri thrips, *Oxythripsagathidis* Morison (Thysanoptera, Thripidae), probe *A.robusta* leaves in Queensland, Australia, and can cause major defoliation (Morison 1941; Heather and Schaumberg 1966).

#### ﻿Mining

A serpentine mine occurs on a single cf. *Agathis* leaf from the Cretaceous Lefipán Formation (Fig. 1H–J), but we did not find similar mines on *Agathis* in any of the Paleogene assemblages. Therefore, this association may have gone extinct regionally, possibly in relation to the end-Cretaceous impact, which caused a decrease in leaf-mining associations in Patagonia and Western Interior North America for ca. 4 and 9 million years, respectively (Wilf et al. 2006; Donovan et al. 2014, 2017, 2018; Labandeira et al. 2002a). Although serpentine mines occur on extant *Agathis* (Fig. 13F, G; Wise 1962), mostly made by unknown insects (Figs 15D–F; 16J, K; 17E, I, K, L, O, P, S; 18E–G, S, V, W), none of the extant mines has strong associations with veins comparable to the Cretaceous specimen (Donovan et al. 2020).

Elongate-ellipsoidal blotch mines occur on *Agathis* in all four fossil assemblages (Figs 1K, 2L–M, 6I, 10C), discontinuously spanning ca. 18 myr. The mines share an overall similar morphology, including an oblong shape, long axis parallel to leaf venation, distorted leaf veins within the mine, and frass composed of spheroidal pellets surrounded by amorphous matter, commonly deposited closer to one mine margin. The mines mainly differ in the structure of their margins (Donovan et al. 2020). *Frondicuniculumflexuosum* mines from PL2 have wrinkled margins that undulate (Fig. 2L, M), and *Frondicuniculumlineacurvum* mines from LH (Fig. 6I) and RP (Fig. 10C) have smooth, gently curving margins. A blotch mine similar to *F.lineacurvum* on cf. *Agathis* from Lef (Fig. 1K) also has smooth, gently curving margins, but lacks frass. The overall similarities between blotch mines on the same genus from the latest Cretaceous and early Paleogene provide the first evidence for a possible Cretaceous–Paleogene boundary-crossing leaf mine association (Donovan et al. 2014, 2017, 2018). Based on a survey of herbarium specimens, at least six extant *Agathis* species covering much of the modern range of the genus are associated with similar blotch mines (Figs 14I–K, 15H, 16C–H, 17J, 18H–L), suggesting possible long-term associations spanning time and space (Donovan et al. 2020).

Putative linear blotch mines with breached epidermal tissue (DT251), described by Donovan et al. (2020), occur in the Eocene at LH (Fig. 6J–L) and RP (Fig. 10DE). Similar damage occurs on *Agathisimmortalis* from the early Paleocene at PL2 (Fig. 2F), although margins of the damage are much less well defined, which may indicate surface feeding. Unlike the breached epidermal tissues characterizing the DT251 mines at LH and RP, the epidermal tissue of the damaged area of the PL2 specimen (Fig. 2F) is apparently intact based on color and texture differences between the damaged areas, undamaged areas, and rock matrix. Morphologically similar leaf mines that are commonly breached due to weathering occur on *Agathisrobusta* in Australia (Fig. 16G, H; Donovan et al. 2020).

#### ﻿Galling

Galls occur on Cretaceous to early Eocene *Agathis* fossils (Lef, PL2, and LH). We found carbonized circular to oval galls (DT32) on cf. *Agathis* leaves at Lef (Fig. 1E) and LH (Fig. 6M), although they lack diagnostic features. At PL2, ellipsoidal to near-circular galls with thickened walls (DT115; Fig. 3A–H) are associated with *A.immortalis*. We did not find DT115 galls on fossil *Agathis* at other localities, but analogous ellipsoidal galls with thickened outer walls surrounding epidermal tissue are associated with *A.ovata* in New Caledonia (Fig. 14L, M; Donovan et al. 2020). Columnar galls typically replaced by or filled in with amber (DT116; Fig. 4) are found exclusively at PL2. These galls, initially defined by Labandeira et al. (2007), share features with the enigmatic structures tentatively interpreted as diaspidid scale covers (Figs 5, 7–9, 11, 12; see discussion below). With the exception of two galls on *A.ovata* and *lanceolata* briefly described by Houard (1914, 1922), documentation of galls on extant *Agathis* is rare, although we found numerous galls on herbarium specimens (Figs 13C–E, 14A, B, H, L, M, 15C, 16B, I, O–Q, 17B–D, G–H, T, U, 18B, C, Q, R, U).

#### ﻿Oviposition

We found oviposition lesions on cf. *Agathis* leaves from the Cretaceous Lefipán Formation (Fig. 1F, G). However, we did not find similar marks on *Agathis* at any other fossil locality or on extant *Agathis* (Donovan et al. 2020). Note added in proof: a single specimen of *Agathiszamunerae* from Laguna del Hunco is associated with an arcuate pattern of ca. 15 subparallel rows of oviposition lesions (DT54; specimen curated at Museo Jorge H. Gerhold (MJHG), Ingeniero Jacobacci, Río Negro, Argentina).

#### ﻿Enigmatic structures: possible armored scale insect (Diaspididae) covers or galls

Enigmatic structures, possibly representing wax coverings constructed by female armored scale insects (Diaspididae) are present on *Agathis* at PL2 (Fig. 5), LH (Figs 7–9), and RP (Figs 11, 12), spanning approximately 16 million years from the early Paleocene to middle Eocene (Suppl. material 1). These structures share many similarities at different fossil sites, including shape and size, concentric growth rings on the dorsal cover, presence of an oval hole or depression near the center of the cover, and a prominent ventral cover (collar). We will first discuss similarities between the enigmatic structures and diaspidid covers and then provide an alternative interpretation of the structures as galls.

The general size and shape of the covers, as well as the presence of concentric rings, are comparable to extant diaspidid covers. The fossil covers are mostly similar in size at all sites but covers at RP are the smallest. The covers are all circular to oval, shapes that tend to be made by female scale insects with near circular bodies (Takagi and Tippins 1972). Interestingly, we found little evidence of first or second instar covers because most of the well-preserved covers with clearly defined growth rings show evidence of three instars. Exceptions include a specimen from PL2 (Fig. 5E) that is associated with varied scale diameters, although growth rings are not preserved. In addition, no evidence for male scale covers was found; as these are usually smaller than the female covers and have an elongate-oval shape (Foldi 1990b), suggesting the likelihood that the possible diaspidids associated with *Agathis* may have been uniparental. Although most diaspidids have sexual mating, some species are hermaphroditic, uniparental (not producing males), or have both uniparental and biparental populations (Miller and Davidson 2005).

Female armored scale insects have three instar stages, including the adult stage. As the insects grow, they add material to their covers, causing an increase in size. The different phases of cover formation can commonly be recognized by distinct rings, indicating the addition of new wax filaments and embedding cement and the incorporation of shed skins (Stoetzel 1976; Foldi 1990b). On well-preserved covers on Paleogene *Agathis* in Patagonia, concentric growth rings are visible on the dorsal covers with prominent rings, indicating distinct instar growth phases. At PL2, scale covers have well-preserved concentric growth rings on some specimens (Fig. 5A–D, I–N), although the transitions between instars are not clear. At LH and RP, impressions (Figs 7C, D, 8H, I) and amber casts (Figs 7H–K, 9F, G, 10) of the covers exhibit distinct growth rings, and some specimens have prominent demarcations of instar transitions.

The surface texture of the dorsal covers varies among fossil localities. At PL2, scale covers are fairly flat and smooth, but the concentric rings are marked with shallow grooves (Fig. 5A). Impressions of scale covers at LH (Figs 7C, D, 8H, I) are also marked with concentric growth rings similar to the covers at PL2 (Fig. 5 I–N), although the transitions between instar stages are much more prominent at LH. However, grooves between growth rings are less prominent on some specimens at LH (Fig. 7H–K), particularly those preserved as amber. Dorsal covers at RP have a slight bumpy surface (Fig. 11), which appears as white round areas under epifluorescence (Fig. 11E, G, I). The RP covers have fewer growth rings overall compared to PL2 and LH, but instar transitions are distinctly visible.

Most dorsal covers at PL2, LH, and RP are associated with a prominent oval, circular, or semicircular hole or impression that is slightly off center. At RP, the edges of the holes are well defined and surrounded by a raised rim (Fig. 11H). The structure is not found on extant diaspidid covers, but possibilities include the position of the white cap constructed by the first instar, which was lost during preservation; the position of unpreserved larval exuviae; or the point where the styletal fascicle was inserted into leaf tissue. Holes on morphologically similar scale covers from the Early Cretaceous of Australia and Late Cretaceous of New Zealand were suggested to have been caused by hymenopteran parasitoids (Tosolini and Pole 2010), which are commonly associated with extant diaspidids and used today as a pest management method (Miller and Davidson 2005). However, the consistent shape, size, and position across the fossil scale-cover morphotypes suggest that parasitoid emergence holes are an unlikely cause. The holes on the fossil covers are positioned along the edges of the first instar covers, but the positions of exit holes on extant scales are less consistent. Every well-preserved dorsal cover is associated with a hole, but usually only a portion of a scale insect population would have been parasitized. Finally, some parasitoid emergence holes have ragged edges, but the holes on the fossils are consistently smooth.

An important shared character between scale covers at PL2, LH, and RP is the presence of a prominent ventral cover (collar), either embedded in leaf tissue (Fig. 7E, H–K) or protruding above the leaf surface (Figs 6, 8J–L, 9C–E). This feature is not directly comparable to features of typical covers made by most extant diaspidids. A possible analog for the construction of the scale covers on fossil *Agathis* is *Cryptaspidiotusbarbusano* (Disapididae: Aspidiotinae), which feeds on *Apolloniasbarbujana* (Lauraceae) in the Canary Islands (Porcelli et al. 2012). After the female molts for the last time, the cover is near circular, flat, and with a slightly conical top (Porcelli et al. 2012). After mating, the adult female constructs a ventral cover, which raises the dorsal cover up, giving the entire scale a cup-like profile. The ventral cover is constructed by the adult female by repeatedly secreting material in a ring pattern (Porcelli et al. 2012). The margins of the ventral cover are raised slightly relative to the dorsal cover at the point where they are connected. Finally, the adult female of *C.barbusano* constructs an exit tunnel for crawlers (Porcelli et al. 2012), which is not found in the fossils.

Female diaspidids on fossil *Agathis* from Patagonia also probably first constructed the dorsal cover during all three instar phases, as evidenced by growth ring patterns. The ventral cover must have been constructed by the adult female after completion of the dorsal cover because it wraps completely around the dorsal cover. The sides of the ventral covers are only visible at LH (Figs 8J–L, 9C–E) and are marked with horizontal and vertical striations (Figs 6H, 9E). The ventral covers are not plant reaction tissue, because they are clearly attached by a joint to the dorsal covers at LH (Fig. 9C) and are composed of the same material as the dorsal covers (amber). There is no evidence of plant cells on the ventral covers, but instead, horizontal striations may indicate where the insects added sequential rings of secretory material. Because ventral covers were constructed last and embedded in plant tissue, first and second instars may not have been strongly attached to the plant. Therefore, they may have been less likely to be preserved, explaining why almost all the intact covers were constructed by third instars.

The Diaspididae (armored scale insects) are the most diverse family of scale insects (Coccoidea), with ca. 2400 known extant species in 380 genera (Miller and Davidson 2005). The taxonomy of Diaspididae is mostly based on morphological characters of adult females (Takagi 1990). The two most common subfamilies of Diaspididae, Aspidiotinae and Diaspidinae, can generally be identified by their cover shape. Typically, aspidiotines produce a circular or oval cover, similar to the fossils on *Agathis*, and diaspidines construct an elongate cover (Miller and Davidson 2005).

Armored scale insects are recognizable by the detachable waxy covering that they construct over their bodies. Female diaspidids have two immature instars before the adult stage, and males have four (first and second instar, prepupal, and pupal). The first instars of both males and females (crawlers) have legs and can disperse, either actively by walking or passively by wind. All other stages are sessile, except for adult males. Adult female diaspidids are characterized by a sac-like body with fused head, thorax, and abdomen, working piercing-and-sucking mouthparts, rudimentary antennae, and absence of legs and wings (Takagi 1990). Posterior abdominal segments of the female are fused into a pygidium with wax glands and anal pores (Takagi 1990). Adult males have a pair of wings and functional legs but lack mouthparts and, therefore, do not feed (Giliomee 1990).

Armored scale insect covers are constructed by all three female instars and the first two out of five male instars. Glands on the pygidium produce wax filaments used to construct the cover, and the anal opening exudes a cementing material (Foldi 1982, 1983, 1990b). Diaspidid species that construct circular or oval scale covers, like the fossils associated with *Agathis*, typically incorporate shed skins in the central or subcentral region of the cover. Detailed treatments of cover formation have been provided in multiple publications (Matsuda 1927; Metcalf and Hockenyos 1930; Dickson 1951; Disselkamp 1954; Foldi 1990a).

The fossil record of Diaspididae is currently rather sparse, with fewer species described than any other scale insect family (Koteja 2000b). The record has been summarized elsewhere (Koteja 1990, 2000a, 2001; Koteja and Ben−Dov 2003; Harris et al. 2007; Wappler and Ben-Dov 2008) and is updated here. Diaspidid covers have been recorded in sediment samples from the Early Cretaceous of Australia and Late Cretaceous of New Zealand (Tosolini and Pole 2010). Aspidiotinae covers attached to angiosperm leaves, including Arecaceae (palms) and unidentified dicots, occur in the middle Eocene of Germany (Wappler and Ben-Dov 2008). Fourteen aspidiotine covers are associated with an angiosperm leaf, possibly Elaeocarpaceae, from the Early Miocene of New Zealand (Harris et al. 2007). *Aspidiotuscrenulatus*, a female body compression fossil found in a Late Miocene deposit from Sicily, Italy, was the first diaspidid fossil described (Pampaloni 1902). Undescribed male specimens occur in middle Eocene Baltic amber (similar to *Lepidosaphes*), middle Miocene Mexican amber, and middle Miocene Dominican amber (Koteja 1990). A putative male diaspidid, *Normarkicoccuscambayae*, was described from the early Eocene of India (Vea and Grimaldi 2015). The species has a short penial sheath compared to extant diaspidid males, which use their elongated penial sheaths to reach female scales under their covers during mating. The difference in morphology suggests the possibility that some female diaspidids from the Eocene or earlier may not have constructed covers (Vea and Grimaldi 2015), although the presence of possible diaspidid covers in sediment samples from the Early Cretaceous (Tosolini and Pole 2010) and unequivocal covers on leaves from the Eocene (Wappler and Ben-Dov 2008) indicates that some members of the family had evolved the behavior.

The possible fossil diaspidid covers associated with Patagonian *Agathis* discussed here are very similar to those reported from the Cretaceous of Australia and New Zealand by Tosolini and Pole (2010). Similarities with the Patagonian fossils include a flat dorsal cover with concentric growth rings and a ventral cover with horizontal and radial striations (Tosolini and Pole 2010), wrinkled folds arranged in a radial pattern (similar to the bumpy ornamentation on the columnar galls on *Agathisimmortalis*; Fig. 4J–L), and an oval hole slightly off center on the dorsal cover (Tosolini and Pole 2010). Tosolini and Pole (2010) noted a possible relationship between the fossil scales and Araucariaceae based on their co-occurrence in Cretaceous beds in a K–Pg boundary section, the lack of scales in Paleocene sediments, and the extinction of some Araucariaceae species at the end of the Cretaceous in New Zealand (Pole 2008; Pole and Vajda 2009). Taken together, these morphologically similar fossils from the Cretaceous of Australia and New Zealand and early Paleogene of Patagonia, if representing diaspidid scale insect covers, suggest that some lineages of Diaspididae had a Gondwanan distribution early in the history of the family and that host-specialization with members of Araucariaceae may have extended outside of Patagonia.

Diaspidids on extant *Agathis* include *Chrysomphalusaonidum* on *A.lanceolata*, *A.moorei* (Brun and Chazeau 1986; Williams and Watson 1990; Mille et al. 2016), and *A.ovata* (Fig. 14N). *Leucaspisportaeaureae* on *A.australis* in New Zealand, *Hemiberlesiarapax* on *Agathis* sp. (Miller and Davidson 2005), and unidentified diaspidids on *A.ovata* in New Caledonia (Fig. 14P) and *A.macrophylla* in Fiji (Fig. 15N, O; Donovan et al. 2020). Females of the unidentified diaspidid species associated with *A.macrophylla* in Fiji induce pit galls (Fig. 15N, O; Donovan et al. 2020), which is the most common method for gall induction in diaspidids (Gullan et al. 2005). A depression is formed around the flat scale cover, and a rim of deformed tissue surrounds the exposed dorsal surface of each cover, similar to the responses of the fossil *Agathis* species. Gall-inducing diaspidids associated with conifers are very rare, and only one other gall-inducing diaspidid has been documented out of 39 species in 28 genera known to induce galls (Gullan et al. 2005). The only other gall-inducing diaspidid on conifers, *Leucaspispodocarpi*, causes leaf margin rolls on *Prumnopitystaxifolia* (Podocarpaceae) in New Zealand (Gullan et al. 2005; Henderson and Martin 2006).

The presence of a gall-inducing diaspidid species on extant *Agathis* (Donovan et al. 2020) is interesting because the possible fossil scales on Patagonian *Agathis* may also have caused similar deformation of the host plant tissue. One *Agathis* specimen at RP is associated with pits (Fig. 12D–G), possibly where covers were positioned. Many other *Agathis* specimens at PL2 (Fig. 5E), LH (Figs 7E, 9I), and RP (Fig. 12A–C) are associated with depressed rims where ventral covers were deeply set. However, we found no evidence of cell hypertrophy or hyperplasia in the areas surrounding the fossil diaspidids.

Other fossil scale insects have shown evidence of host deformation. Diaspidid (Aspidiotinae) scale covers on dicotyledenous leaves from the middle Eocene of Germany are surrounded by a ring of raised tissue, presumably a reaction to insect feeding (Wappler and Ben-Dov 2008). Depressed circular to oval rings on primary veins of *Erlingdorfiamontana* (Platanaceae) from the Late Cretaceous (Maastrichtian) of North Dakota, USA were possibly made by an unidentified coccoid (Labandeira et al. 2002b).

Although the structures share many aspects of general morphology with diaspidid covers (size, shape, concentric growth rings, low domal structure), some features of the Patagonian fossil structures differ from those associated with extant scales. One, the prominent hole, called a parasitoid emergence hole in Tosolini and Pole (2010), is not a normal feature of modern scale insects. The size, shape, and position of the holes are much more consistent than typical parasitoid emergence holes. Secondly, the sizes of covers on a leaf are consistent. If they were scale covers, we would expect to see differences in size representing different larval growth stages and cover sizes, unless early instars were not strongly attached to the plants and not preserved or alternatively these were new populations of diaspidids. The only specimen with notable differences in sizes among individual scales is on *Agathisimmortalis* from PL2 (Fig. 5E). In addition, there is no evidence of sexual dimorphism in scale covers. Finally, the collar-like structure of the ventral cover, which surrounds the scale covers, is atypical for most extant scales.

A second possible explanation for the enigmatic structures is galling. There are many similarities between the fossil columnar gall at PL2 (DT116; Fig. 4) and the possible diaspidid scale covers. Both the galls and covers have a “collar” (ventral cover) marked by vertical striations (Figs 4F–H, 9C–E). However, the “collars” tend to wrap around the top of the galls (Fig. 4J), instead of neatly encircling the dorsal covers (Fig. 9C). In addition, some of the galls have a center to off-center hole (Fig. 4J–L), interpreted as an exit hole, fungal ostiole, or standard shape of the gall, which corresponds to a similar structure on the covers (Figs 5L, 7H–K, 11B–I). Both the galls and covers are preserved as amber. Concentric rings on the galls, if present, are vaguely defined, and marked by rounded bumps or pointy ornamentation (Fig. 4J–L). Dorsal covers at RP have a slightly bumpy texture (Fig. 11D), which may correspond to the ornamentations on the galls. Alternatively, DT116 at PL2 may not be a gall, but instead a preservational form of a scale cover that has been altered in some way before fossilization. Overall, the similarities between the fossil galls and covers, in addition to the atypical features discussed in the previous paragraph, complicate the interpretation of the structures as being made by diaspidids. Although their origin remains inconclusive, the enigmatic structures demonstrate persistence across an impressive spatial and temporal range, occurring on early Paleocene–middle Eocene *Agathis* in Patagonia (Donovan et al. 2020) and in sediment samples from the Cretaceous of Australia and New Zealand (Tosolini and Pole 2010).

## ﻿Rust fungus (Pucciniales)

Rust fungi (Pucciniales) are obligate parasites associated with ferns, gymnosperms, and angiosperms. Their common name comes from the spores that they produce, typically yellow or orange, which germinate on plant hosts, leading to a rust-like appearance. Rusts can cause deformation of plant hosts in various ways, including inducing galls, witch’s brooms, and cankers. Extant *Agathis* hosts diverse fungal communities (McKenzie et al. 2002), including two species of the recently named rust fungus genus *Araucariomyces* (Araucariomycetaceae; the species were formerly placed in *Aecidium*; Aime & McTaggart, 2021), which parasitizes *Agathis* through much of its range. *Araucariomycesfragiformis* (previously *Aecidiumfragiforme*; Fig. 15K) is associated with *Agathis* in Australia, the Solomon Islands, Fiji, Vanuatu, New Guinea, Borneo, and Malaysia, and *Araucariomycesbalansae* (previously *Aecidiumbalansae*) is restricted to New Caledonia (Punithalingam and Jones 1971; McKenzie et al. 2002). Aeciospores can travel long distances by wind, which probably facilitated *A.fragiformis* to track *Agathis* through a large portion of its extant range. *Araucariomyces* on extant *Agathis* produce galls covered in yellow aecia with pycnia embedded in the opposite surface (Peterson 1968; Punithalingam and Jones 1971). The species are differentiated by the morphology of the aeciospores. The surfaces of aeciospores produced by *A.balansae* are covered in coarse warts, and the surfaces of aeciospores produced by *A.fragiformis* are covered in spines (Punithalingam and Jones 1971). Aecia tend to be deeply-set in swollen tissue on the abaxial leaf surface for *A.balansae* and the adaxial leaf surface for *A.fragiformis* (Punithalingam and Jones 1971). Although rusts on plant species co-occurring with *Agathis* in Australasia and Southeast Asia have been sampled extensively, the telial state for both *Araucariomyces* species is unknown, leading to speculation that *Araucariomyces* may not have a sporothallus stage and instead systemically infects *Agathis* (Aime and McTaggart 2021). Recent phylogenetic analyses of Pucciniales show *Araucariomyces* as a separate lineage from the rest of the order (Aime and McTaggart 2021). The morphological similarity between the fossil rust fungus on *Agathiszamunerae* from LH and *A.fragiformis* and *A.balansae* on extant *Agathis*, including concentric rings of deep-set aecia in swollen leaf tissue, suggests the possibility of ancient coevolutionary relationships between these groups (Donovan et al. 2020).

The evolutionary history of rust fungi is poorly understood (Tiffney and Barghoorn 1974; Pirozynski 1976). The earliest probable rust, Teleutosporites (Uromyces), is associated with *Lepidodendron*, an extinct representative of lycopsids, from the Pennsylvanian subperiod (Renault 1893; Tiffney and Barghoorn 1974). The earliest rust accepted by Tiffney and Barghoorn (1974), *Puccinites*, is associated with a monocot leaf from the Eocene of western Tennessee (Dilcher 1965). Past estimates of the most recent common ancestor of rust fungi vary between 300 to 113 Ma, and whether the main driver of rust diversification was convergence and coevolution with major plant lineages, or host switching, or a combination of both is debated (Leppik 1965; Savile 1976; Wingfield et al. 2004; Aime 2006; McTaggart et al. 2016; Aime et al. 2018). The relationships between plant hosts of rust fungi gametothalli, the stage visible on the fossil, appear to be related to the systematic relationships of Pucciniales, highlighting the role of coevolution and biological specialization in the diversification of Pucciniales (Aime et al. 2018). Fossil spores, not preserved here, are needed for finer taxonomic resolution of the fossil rust fungus on *Agathiszamunerae* (Fig. 6N–P). However, based on overall morphological similarity to *Araucariomyces* species on extant *Agathis*, the fossil fungus suggests possible long-term coevolutionary relationships between the aecial stage of a rust fungus and its host genus.

## ﻿Persistence of plant-insect interactions on *Agathis* through time

Ecological guilds and possibly herbivore communities on Patagonian fossil *Agathis* exhibit remarkable host-fidelity and evolutionary conservatism across sites through time, during the process of evolving modern characteristics of the genus during the Cretaceous and early Paleogene (Wilf et al. 2014; Escapa et al. 2018). Persistence of these ecological guilds also occurred through major environmental changes, such as the Cretaceous–Paleogene extinction, early Eocene warming (Wilf et al. 2003, 2005b; Donovan et al. 2017), and 45 million years of post-Gondwana events. Most strikingly, similar enigmatic structures, possibly covers made by armored scale insects (DT86) or galls, occur in all early Paleogene assemblages, linear blotch mines with breached epidermal tissue (DT251) occur in both Eocene assemblages, and elongate-ellipsoidal blotch mines occur in all assemblages (DT88 and DT421); Donovan et al. 2020), crossing the Cretaceous-Paleogene boundary. Associations that are unique to one time period, suggesting possible extirpation, extinction, or undersampling, include serpentine mines influenced by leaf veins (DT139) and elliptical oviposition marks (DT101) at LefE.

Persistent associations on latest Cretaceous to early Paleogene fossil *Agathis* tend to have extant analogs on *Agathis* in Australasia to Southeast Asia, raising the possibility that some of these associations may have tracked the genus through major range shifts, continental breakup, and environmental change (Donovan et al. 2020). Possible armored scale insects associated with early Paleogene *Agathis* caused deformation to the host tissue, leaving pits or depressed rims (DT86; Fig. 8E). On *A.macrophylla* in Fiji, a diaspidid causes host tissue deformation by inducing pit galls on the leaves (Fig. 15N, O; Donovan et al. 2020). Linear blotch mines with breached epidermal tissue (DT251; Figs 6J–L, 10D, E) from the early Eocene assemblages resemble mines on *A.robusta* in Queensland, Australia. Elongate-ellipsoidal blotch mines (DT88; Figs 1K, 6L, M, I, 10C) on *Agathis*, described by Donovan et al. (2020), span the latest Cretaceous to early Paleogene, and similar mines are associated with eight species of extant *Agathis* through much of its range. External foliage feeding, such as hole feeding (DT1; Fig. 1A, 2A, B, 6A), margin feeding (DT12; Figs 1B, 2C, D, 6C–F, 10A), and surface feeding (DT29; Figs 1C, 2F, 6G) are found on *Agathis* at multiple Patagonian fossil localities and are common on extant *Agathis*, although similarities in these damage morphologies do not necessarily imply common tracemakers (Carvalho et al. 2014). The pattern of persistence to the modern day observed in the previous examples was not always found for associations that only appeared during a single time slice, such as a serpentine mine (Fig. 1H–J) or oviposition lesions (Fig. 1F, G) from the latest Cretaceous, for which we have not found clear modern analogs. Exceptions include the rust fungus fossil, found on one *A.zamunerae* leaf from the early Eocene (LH; Fig. 6N–P), which is similar to two species of *Araucariomyces* associated with extant *Agathis*. Overall, most of the extant associations we found on extant *Agathis* are previously undocumented, and the insects that made the damage are unknown. Consequently, taxonomic and ecological observations of the extant plants and insects are needed to understand these patterns first observed in the fossil record.

The persistence of plant-insect associations over geologic timescales has previously been observed in the paleobotanical record (Opler 1973; Labandeira et al. 1994; Wilf et al. 2000; Winkler et al. 2010; Leckey and Smith 2015; Su et al. 2015; Adroit et al. 2020), although the recurring presence of multiple components of the insect herbivore and fungal communities on a single genus for millions of years and into the modern day is rare (Donovan et al. 2020). Other examples of persistent associations include surface feeding damage made by hispine beetles on Zingiberales fossils from latest Cretaceous and Eocene deposits in Western Interior North America, an association that still occurs in the modern Neotropics (Wilf et al. 2000). On oaks, a minimum Miocene age has been suggested for eleven leaf mines based on morphological similarity between fossil and extant mines (Opler 1973), galls similar to those made by Cynipini wasps are found associated with two oak species from the Oligocene to Pliocene (Leckey and Smith 2015), and nearly all DTs on oak leaves from a Pliocene fossil assemblage in southwestern China occur on extant oaks in local forests (Su et al. 2015). Distinct curvilinear zones of skeletonized tissue have been associated with *Parrotia* (Hamamelidaceae) leaves for at least 15 million years, from the Miocene of China and Pliocene of Germany to modern Iran and China (Adroit et al. 2020). The study of fossil plant-insect associations is necessary to address fundamental issues concerning patterns of insect host use over time, including the prevalence of evolutionarily conservative, long-term associations (Labandeira et al. 1994; Wilf et al. 2000; Labandeira 2002; Labandeira and Wappler 2023), host-switching (Labandeira 2002), and extinct associations (Labandeira 2002; Winkler et al. 2010).

The case study of *Agathis* and its herbivore communities presented here and earlier (Donovan et al. 2020) provides some insights into potential causes of persistent interactions between plants and associated insects and fungi. *Agathis* appears to be conservative in both its morphology and habitat preferences (Kooyman et al. 2014; Wilf et al. 2014), tracking rainforest environments throughout its history. Niche conservatism, the tendency for closely related species to occupy similar niches (Pyron et al. 2015), has been observed at local and regional scales (Losos et al. 2003; Silvertown et al. 2006), and across continents (Crisp et al. 2009). Tracking of everwet rainforest biomes appears to be a common pattern for extant members of many of the plant groups present in early Paleogene fossil deposits from Patagonia (Kooyman et al. 2014; Merkhofer et al. 2015). The environmental stability caused by plant biome tracking may have provided suitable environments for ecological guilds and possibly herbivore communities, including those on *Agathis*, to establish long-term, coevolutionary relationships with their hosts (Donovan et al. 2020) that are now endangered due to habitat loss and climate change (Kooyman et al. 2022).

*Agathis* has evolved numerous defenses against insect herbivores. *Agathis* leaves are tough and leathery, contain copious resin (Langenheim 1990, 1996), tannins (Arbicheva and Pautov 2018) and phenolic compounds (Gadek et al. 1984), and are coated with epicuticular waxes (Dragota and Riederer 2008). When injured, *Agathis* leaves produce true wound periderm (Arbicheva and Pautov 2018). *Agathisrobusta* leaves produce stomatal wax plugs, which have been shown experimentally to block fungal hyphae from entering stomatal pores (Mohammadian et al. 2009).

The presence of *Agathis* in Patagonia before the final breakup of Gondwana (Wilf et al. 2014; Escapa et al. 2018) suggests that the genus probably reached its extant range through a combination of vicariance and dispersal over water. Several plant groups represented by fossils at Laguna del Hunco are older than recent molecular clock estimates, underscoring the importance of fossils for understanding Gondwanan legacies in modern distributions (Wilf and Escapa 2015). Insect herbivores on *Agathisaustralis* in New Zealand are better documented than on *Agathis* in other regions. However, distinct insect damage on *A.australis*, such as mines made by *Parectopaleucocyma* (Wise 1962), have not been found in other parts of the extant range of the genus or on fossils (Donovan et al. 2020). Notably, Donovan et al. (2020) did not find any types of blotch mines on *A.australis*, and *Araucariomyces* rust fungus is not associated with *A.australis* in New Zealand despite the wide range of the association (McKenzie et al. 2002). *Agathis* fossils have been described from the late Paleocene to early Miocene of Australia and the late Oligocene–Miocene of New Zealand (Hill et al. 2008; Pole 2008). Insect damage has not been reported on *Agathis* and *Agathis*-like fossils from Australia and New Zealand, and besides a possible gall on *Agathis* sp. aff. *A.robusta* from the middle Miocene of New South Wales, Australia (Holmes and Andereson 2019), we did not observe any insect damage on published images of fossil *Agathis* leaves from these regions (Cookson and Duigan 1951; Hill and Bigwood 1987; Cantrill 1992; Hill and Merrifield 1993; Pole et al. 1993; McLoughlin and Hill 1996; McLoughlin et al. 2001; Hill et al. 2008; Pole 2008; Holmes and Andereson 2019). Future discoveries of well-preserved *Agathis* macrofossils with insect damage from the current range of the genus may provide further insight into the biogeographic history of phytophagous insects associated with *Agathis*.

## ﻿Conclusions

Our documentation of fossilized *Agathis* herbivore communities from the latest Cretaceous to middle Eocene of Patagonia illustrates several persistent forms of damage, including external foliage feeding, leaf mines, enigmatic structures (possibly scale insect covers or galls), and a rust fungus. *Agathis* remains an important host genus for insect herbivores and pathogens today, as evidenced by the diverse array of damage that we found on 15 extant species across their Australasian and Southeast Asian range. Most of the extant damage on *Agathis* is undescribed and much of it is similar to the fossils, demonstrating the importance of integrating fossil and extant plant-insect associational data to explore long-term evolutionary and ecological patterns of host-plant use.

## Supplementary materials

Supplementary material 1 Descriptions of DT421 (new damage type), DT86, and DT116 Data type word file Explanation note Descriptions of blotch mines (new damage type DT421), enigmatic structures, possibly armored scale insect (Diaspididae) covers (DT86), and columnar galls (DT116). https://binary.pensoft.net/file/855015This dataset is made available under the Open Database License (http://opendatacommons.org/licenses/odbl/1.0/). The Open Database License (ODbL) is a license agreement intended to allow users to freely share, modify, and use this Dataset while maintaining this same freedom for others, provided that the original source and author(s) are credited.Michael P. Donovan, Peter Wilf, Ari Iglesias, N. Rubén Cúneo, Conrad C. Labandeira
