# Supplementary material for: ﻿Insect herbivore and fungal communities on Agathis (Araucariaceae) from the latest Cretaceous to Recent
Source: PhytoKeys. 2023 May 26;226:109–58. doi: 10.3897/phytokeys.226.99316 (PMC10239022; doi:10.3897/phytokeys.226.99316)
Supplement: Supplementary material 1 — Descriptions of DT421 (new damage type), DT86, and DT116 [file phytokeys-226-109_article-99316__-s001.docx]

Supplementary Information

**Description of new damage type DT403.**

We define DT403 for use in future versions of the *Guide to Insect (and Other) Damage Types on Compressed Fossil Plants* (Labandeira *et al.*, 2007). Short and long descriptions of DT403 are modified from the genus diagnosis and species descriptions of *Frondicuniculum flexuosum* in Donovan *et al.*, 2020.

**DT403. MINING.**

**Short description:** Elongate-ellipsoidal blotch mine with wavy mine margins occurring on broadleaved, parallel veined leaves.

**Long description:** Elongate-ellipsoidal blotch mine with gentle to strongly undulatory margins having a raised, wrinkly appearance. Mine positioned along leaf margin, long axis of the mine parallel to leaf veins. Frass, when present, composed of spheroidal pellets measuring ca. 0.1 mm in diameter and surrounded by smaller fragments of amorphous frass. Frass distributed throughout mine or positioned laterally near one margin of the mine. Texture of the internal mine may be characterized by a subtly transversely oriented, bulbous surface.

**Ichnotaxonomy**: *Frondicuniculum flexuosum*

**Feeding event occurrence data:** Single

**Damage type functional breadth:** 3 = narrow

**Plant host***: Agathis immortalis* (Araucariaceae)

**Locality**: Palacio de los Loros 2, Chubut Province, Argentina

**Stratigraphy**: Salamanca Formation

**Age**: Paleocene, Danian

**DT geochronologic range**: Cretaceous-Recent

**Specimen**: MPEF-Pb 5970

**Repository**: Museo Paleontológico Egidio Feruglio, Trelew, Chubut, Argentina

**Inferred herbivore**: Unknown

**Modern ecological analog**: *Peristoreus flavitarsis*, the totara leafminer weevil, mines *Podocarpus totara* (Podocarpaceae) leaves in the North and South Islands of New Zealand (Martin, 2018). A number of blotch mines of unknown origin with similar morphologies to DT403 are associated with extant *Agathis* in Australasia and Southeast Asia (Donovan *et al.*, 2020).

**References**

Donovan MP, Wilf P, Iglesias A, Cúneo NR, Labandeira CC (2020) Persistent biotic interactions of a Gondwanan conifer from Cretaceous Patagonia to modern Malesia. Communications Biology 3: 708. https://doi.org/10.1038/s42003-020-01428-9

Labandeira CC, Wilf P, Johnson KR, Marsh F (2007) Guide to Insect (and Other) Damage Types on Compressed Plant Fossils. Version 3.0. Smithsonian Institution, Washington, D.C., 25 pp. Available from: http://paleobiology.si.edu/insects/index.html.

Martin NA. (2018) Totara leafminer weevil—*Peristoreus flavitarsis*. Interesting insects and other invertebrates. N. Z. Arthropod Factsheet Series Number 140.

**Descriptions of enigmatic structures, possibly armored scale insect (Diaspididae) covers, and columnar galls (DT116)**

*Palacio de los Loros 2*

*DT86*

MPEF-Pb 6096 (Fig. 5A**–**D) – Two covers marked by approximately 15**–**20 concentric growth rings and preserved as amber casts. The covers measure 1.45**–**1.62 mm in diameter. An oval is located on the possible first instar cover (Fig. 5D) and measures 0.20 mm long by 0.13 mm wide. Concentric growth rings are 0.02**–**0.03 mm apart from each other. Boundaries representing the transitions from first to second instar and adult are not discernable. The covers are surrounded by a ventral cover measuring 0.08**–**0.09 mm wide.

MPEF-Pb 6113 (Fig. 5E) – Approximately fifteen densely packed cover impressions with little detail preserved. The ventral covers are depressed into the leaf and the central region is flat or slightly domed. The covers are circular and measure between 0.6**–**2.1 mm. The wide variation in diameters suggests the presence of multiple instars and intra-instar growth increments.

MPEF-Pb 6020 (Fig. 5F, G) – Sixteen circular to oval covers clustered together near the base of the leaf preserving only the deeply embedded ventral covers. The smallest cover measures 1.1 by 0.7 mm, and the largest measures 1.5 by 1.3 mm. The ventral covers measure 0.1 mm wide (Fig. 5G). Some ventral covers of different scales touch each other, causing deformation of the typical circular or oval shape.

MPEF-Pb 5861 (Fig. 5H) – Oval cover attached to the upper edge of an *Agathis* cone scale. The cover measures 1.5 mm long by 1.1 mm wide with a 0.11**–**0.12 mm wide ventral cover. Both the ventral and dorsal covers are preserved as amber, but no details of the dorsal cover, such as growth rings, are preserved. The ventral cover is raised above the leaf surface.

MPEF-Pb 5996 (Fig. 5I**–**M) – Three examples of scale insect covers marked by concentric growth rings. The scales are circular and measure 1.50**–**1.65 mm in diameter. The approximately 17 concentric rings are positioned 0.04**–**0.08 mm apart and are generally more closely spaced near the center of the cover (Fig. 5L). Although concentric rings are clearly visible, different instar and adult growth stages are difficult to discern because particularly pronounced concentric rings are not present. An oval bump positioned at the edge of the first instar cover (Fig. 5I, K, L), possibly remnants of the first instar exuviae or stylet fascicle for feeding on host tissue below. This structure measures 0.17**–**0.19 mm long by 0.14**–**0.16 mm wide.

PL2 331 – Four structures characterized by a deeply set ventral cover surrounding a flat to slightly domed center with no details of the dorsal cover preserved.

PL2 2005 450 – Approximately circular scale measuring 1.4**–**1.5 mm in diameter, preserving only the deep-set ventral cover (0.1 mm wide). The scale is positioned along the leaf margin. No detail of the dorsal cover is preserved in the central area.

PL2 2005 1225 – Approximately circular cover preserved as an amber cast measuring 0.9 mm maximum diameter by 0.77 mm minimum diameter. Details of cover growth associated with two instars and the adult phase are preserved as concentric rings, which are positioned approximately 0.2 mm apart. The scale is located about 0.2 mm away from the leaf margin.

PL2 2005 1463 – Circular cover measuring 1.2 mm in diameter. The ventral cover is deeply set in the leaf, protruding above the leaf surface, and the inside is filled with sediment. The ventral cover is composed of two concentric rings measuring 0.12 mm wide combined. The cover is located 1.3 mm away from the leaf margin.

MPEF-Pb 5875 – Eleven poorly-preserved covers. They are oval-shaped, measuring 1.4**–**1.6 mm long by 1.3**–**1.5 mm wide, and their long axes tends to be parallel to the leaf veins. One of the scales is preserved as a dark brown amber cast with faintly preserved concentric growth rings.

MPEF-Pb 5985 – Six or more approximately circular scales. Only two scales, measuring 1.5**–**1.7 mm in diameter, preserve the ventral covers, which measure 0.06**–**0.08 mm wide. The rest of the scales are characterized by either a circular dome or a depressed ring where the ventral cover was situated. The central portions of the scales, where the dorsal cover would have been located, have no discernable structures.

MPEF-Pb 5989 – Approximately circular scales only preserving the deep-set ventral covers. The covers are located along the central axis of the leaf. The scale positioned closer to the leaf apex measures 1.19 mm long by 1.00 mm wide, and the cover located closer to the base of the leaf measures 1.62 mm long by 1.38 mm wide. The width of the ventral covers is 0.06**–**0.13 mm, and the centers are infilled with sediment with faint evidence of concentric growth rings.

MPEF-Pb 5997 – Eleven or twelve circular scales preserved as impressions of the ventral covers surrounding flattened or slightly domed sediment. The scales measure 0.6**–**1.4 mm in diameter, and the ventral covers are 0.2 mm wide. Some of the ventral cover depressions have remnants of the covers represented by thin layers of amber.

MPEF-Pb 6061 – A single scale composed of a ventral cover embedded into the leaf tissue and surrounding the dorsal scale cover. The ventral cover is composed of amber, but no details of the dorsal cover were preserved. The scale is oval, measuring 1.5 mm maximum diameter by 1.3 mm minimum diameter, and the ventral cover measures approximately 0.1 mm wide.

*DT116*

MPEF-Pb 6023 (Fig. 4A**–**D) – Five deep-set galls. Two of the galls are deeply-set and protrude from the leaf and surrounding infilled sediment (Fig. 4B, C). The diameters of the galls are 1.2**–**1.3 mm. The upper rims of the sides curve slightly over the edge of the infilled sediment. The other three galls are represented by pits where the galls were originally positioned and subsequently detached (Fig. 4D). These gall impressions measure 1.2**–**1.8 mm in diameter.

MPEF-Pb 5995 (Fig. 4E**–**H) – A gall measuring 1.3 mm in diameter. The side collar-like structure curves slightly over the edge of the top of the gall (Fig. 4E) and is characterized by horizontal and vertical striations (Fig. 4F). The collar measures 0.02**–**0.04 mm wide and protrudes 0.35**–**0.39 mm above the surface of the leaf. The top of the gall has a small circular bump (0.2 mm diameter) near the center. When the gall is viewed under epifluorescence, beadlike structures are visible (Fig. 4H). On the counterpart, five galls are represented by shallow impressions measuring 1.1**–**1.9 mm in diameter with 0.2 mm wide collars.

MPEF-Pb 5960 (Fig. 4I) – Galls ornamented by rounded bumps arranged in concentric rings. The gall measures 1.0 mm in diameter and is surrounded by a horizontally and vertically striated collar, which measures 0.05**–**0.12 mm wide. A circular hole near the center of the gall measures 0.12 mm in diameter and may represent the exit hole. The top of the gall is raised above the leaf surface and consists of an amber cast.

MPEF-Pb 9750 (Fig. 4J**–L**) – Amber casts of galls measuring 0.89**–**1.05 mm maximum diameter by 0.77**–**0.99 mm minimum diameter. The galls are ornamented with botryoidal to pointy protrusions arranged in concentric rings. An oval hole, possibly representing an exit hole, is present near the center of most of the galls and measure 0.22**–**0.23 mm long by 0.17**–**0.21 mm wide. The galls are surrounded by collars that wrap partially over the tops of the galls. The collars are marked with horizontal and vertical striations spaced 0.02**–**0.03 mm apart.

PL2 277 – Eight or more galls composed of an external collar surrounding a flattened or domed center. The galls are circular to oval and measure 1.1**–**1.8 mm in diameter. The collars are raised relative to the tops of the galls and are characterized by vertical and horizontal striations.

PL2 2005 349 – Circular gall measuring 1.1 by 0.9 mm in diameter. Only the amber collar is preserved, which measures 0.1 mm wide.

MPEF-Pb 6065 – Collars surrounding infilled sediment protruding from a leaf. The diameter of the gall is 0.9**–**1.0 mm. Under fluorescence, beadlike structures are visible. The collar is characterized by horizontal and vertical striations.

*Laguna del Hunco*

MPEF-Pb 6307 (Fig. 7A**–**D)– Eleven scale covers on a branch and associated leaves. The scale covers are oval in shape and measure 1.42**–**1.63 mm in length by 1.25**–**1.45 mm in width. Each scale is characterized by concentric rings with more pronounced rings representing cover growth during the two instar and adult phases of diaspidid females (Fig. 7C, D), the latter of which has about 15 rings. The first instar cover measures 0.47**–**0.51 mm in length by 0.35**–**0.40 mm in width, increases to 0.71**–**0.92 mm in length by 0.68**–**0.73 mm in width during the second instar phase, and the adult cover measures 1.35**–**1.47 mm in length by 1.17**–**1.33 mm in width. The ventral cover measures 0.04**–**0.10 mm wide.

MPEF-Pb 6349 (Fig. 7E) – Single scale represented by an approximately circular, depressed rim where the ventral cover was situated. The diameter of the area enclosed by the rim measures 1.40 mm long by 1.25 mm wide, and the depressed rim measures 0.10**–**0.15 mm wide. No details of the dorsal cover were preserved in the center region.

MPEF-Pb 6360 (Fig. 7F) – Two pits near the leaf apex possibly representing depressions where scale insects were feeding. One pit is circular, measuring 0.96 mm in diameter, and the second pit measures 0.71 mm long by 0.63 mm wide. No details of the scale covers were preserved.

MPEF-Pb 6383 (Fig. 7G**–**K) – Six well preserved scale covers surrounded by raised ventral covers. Scale covers measure 0.9**–**1.0 mm in diameter and are characterized by linear radiations, and more prominently concentric rings representing cover formation associated with larval growth. The first instar cover measures 0.38**–**0.42 mm in length by 0.31**–**0.38 mm in width, increases to 0.58**–**0.62 mm in length by 0.62**–**0.63 mm in width during the second instar phase, and grows to 0.70**–**0.85 mm in diameter during the adult phase. The ventral covers measure 0.07**–**0.15 mm wide. An oval hole (Fig. 7I, K), possibly representing the position of exuviae or the styletal ensemble, is found on some of the dorsal covers and measures 0.22 mm in length by 0.14 mm in width. The scales are surrounded by a black reaction rim measuring 0.09**–**0.13 mm wide.

MPEF-Pb 6363 – Three pits possibly representing where scale insects were positioned. The three pits are oval, measuring 1.5 mm long by 1.0 mm wide, 1.7 mm long by 1.1 mm wide, and 0.8 mm long by 0.7 mm wide, respectively. Darkened rims surround the pits measure 0.1**–**0.2 mm wide. Leaf veins are visible within pits and the long axes of the pits are parallel to the leaf veins.

MPEF-Pb 6324 (Petru 1) (Fig. 8) – Approximately 37 scale covers on a branch and associated leaves. Many of the scales are positioned in a linear row down the center of one of the leaves (Fig. 8B). The scale covers measure 1.20**–**1.46 mm diameter. On scales where it is visible, the first instar dorsal cover measures 0.51**–**0.57 mm in length by 0.41**–**0.44 mm in width, grows to 0.84**–**0.86 mm long by 0.75 mm wide during the second instar, and increases to 1.1**–**1.30 mm in length by 1.1**–**1.17 mm in width during the adult phase (Fig. 8H, I). The ventral covers measure 0.04**–**0.05 mm wide (Fig. 8D). An oval hole or bump or pustule, possibly representing the position of exuviae or the stylet housing, is found on some dorsal covers and measures 0.24 mm long by 0.18 mm wide (Fig. 8G**–**I). On scales that do not have concentric growth rings on the dorsal cover, the internal features are characterized by beadlike structures (Fig. 8G). Some scales are positioned close and occasionally impinge on each other, causing deformation of their typical oval shape. The central cover has less relief than the outer ventral cover, usually around 0.4 mm lower in height (Fig. 8J, K). Visible ventral cover height is around 0.21**–**0.26 mm (Fig. 8L), although it appears that the ventral covers are deeply-set into the leaf tissue.

MPEF-Pb 9842 (Fig. 9) –Nine scale covers on a branch and associated leaves (Fig. 9A, H). The covers are preserved as amber casts of the dorsal covers and ventral covers (Fig. 9C**–**G), impressions of dorsal covers with amber ventral covers (Fig. 9B), and depressed rims where the ventral covers were located (Fig. 9I, J). Dorsal covers are 0.89**–**1.45 mm in diameter and surrounded by 0.11**–**0.15 mm wide ventral covers. Two ventral covers have been uplifted and are exposed in side view and measure 0.55**–**0.75 mm in height (Fig. 9C**–**E). The ventral covers are marked with horizontal and vertical striations, spaced 0.02**–**0.04 mm and 0.03**–**0.05 mm apart, respectively.

Río Pichileufú

USNM 545228 (Fig. 11) – A leaf with many scale covers of which at least half are positioned over major veins. The scale covers are approximately circular and measure 0.70**–**84 mm with a mean diameter of around 0.75 mm. Details of the growth patterns of the dorsal covers are indicated by concentric rings, which exhibit evidence of two instars and an adult phase (Fig. 11D**–**G, I, K). First instar covers measure 0.40x0.38 mm, increase to 0.53**–**0.55 by 0.55**–**0.57 mm during the second instar phase, and measure 0.66 by 0.71**–**0.72 mm during the adult stage. An oval hole (Fig. 11D**–**G, I, K), possibly representing the position of exuviae or styletal ensemble, is found on the edge of most of first instar portions of the dorsal covers. The oval holes measure 0.22**–**0.23 mm in length by 0.15**–**0.16 mm in width and are surrounded by a rim measuring 0.02**–**0.06 mm wide (Fig. 11H). Under epifluorescence, patterns circular to ellipsoidal marks are visible throughout the scales (Fig. 11E, G, I). The marks measure 0.01**–**0.02 mm in diameter and tend to decrease in size as development occurs from the first instar to second instar to adult growth stages of the cover. The thickness of the cover also appears to decrease after each successive growth stage. The dorsal covers are surrounded by a ventral cover measuring 0.02**–**0.06 mm in width and is deeply embedded in the leaf tissue.

USNM 545223 (Fig. 12A**–**C) – Five approximately circular scales are represented by depressed rims where the ventral covers were originally positioned. No details of the dorsal covers were preserved. The overall diameter of the scales is 0.98**–**1.08 mm and the ventral covers measure 0.5**–**0.9 mm wide. Two scales are positioned along the central axis of the leaf and three are near the leaf margins.

USNM 545226 (Fig. 12D**–**G) – Circular pits in the leaf which may represent scale positions. However, these pits are mostly smaller than scale covers on other leaves. The shallow pits (Fig. 12D) measure 0.65**–**0.86 mm in diameter and are surrounded by rims of sandy material and with slight relief. Deeper pits (Fig. 11E**–**G) measure 0.32**–**0.41 mm in diameter and exhibit reaction rims measuring 0.9**–**0.17 mm wide. All of the pits are located on a blotch mine.

USNM 545224 – A circular impression may indicate the position of scale insect feeding. The pit measures 0.80**–**0.92 mm in diameter, and a surrounding lighter-colored rim measures 0.06**–**0.16 mm wide. No evidence of the ventral cover or dorsal cover was preserved.
